# Revealing Functional Traits of Insect Pest Suppressive Rhizobacterial Strains Through Comparative Genomics

**DOI:** 10.1111/1462-2920.70409

**Published:** 2026-09-07

**Authors:** Edward C. Cairns, Francesco Del Carratore, James P. J. Hall, Sharon E. Zytynska

**Affiliations:** ^1^ Department of Evolution, Ecology and Behaviour, Institute of Infection, Veterinary and Ecological Sciences University of Liverpool Liverpool UK; ^2^ Department of Biochemistry, Cell and Systems Biology Institute of Systems, Molecular & Integrative Biology, the University of Liverpool Liverpool UK; ^3^ Department of Earth and Environmental Sciences, Faculty of Science and Engineering The University of Manchester Manchester UK

## Abstract

Root inoculation with rhizobacteria is an emerging strategy to enhance plant resistance to aphid herbivory, yet the microbial functional traits underpinning these responses remain poorly characterised. Here, we present a comparative genomic analysis of five rhizobacteria ( *Acidovorax radicis* N35, *Bacillus subtilis* B171*, Bacillus velezensis* FZB42*, Rhizobium radiobacter* F4 and *Pseudomonas simiae* WCS417r) that suppress aphids when inoculated onto barley. As expected, functional variation largely reflected phylogenetic relatedness; however, candidate traits implicated in modulation of plant immune defences were conserved across all strains, including biosynthesis of 2,3‐butanediol, riboflavin and salicylic acid. Additional shared functions, linked to plant defence signalling, included phytoene and squalene biosynthesis (absent in *P. simiae* ) and N‐acyl homoserine lactone quorum sensing (absent in *Bacillus* spp.). Strain‐specific traits were also identified, including surfactin production in *Bacillus* spp. and hydrogen cyanide biosynthesis in *A. radicis* and *P. simiae* . Comparison with a broader collection of rhizobacteria revealed that many putative plant‐beneficial functions identified were widely conserved, including among closely related phytopathogens. This extensive functional overlap suggests aphid suppression cannot be explained solely by presence or absence of broad functional traits, but rather by specific trait combinations, regulatory differences, or context‐dependent expression. This highlights the need for genome‐informed approaches for bioinoculant discovery.

## Graphical abstract

Inoculation of select rhizobacterial strains to enhance crop health is a growing field in sustainable agriculture. This study conducts a comparative genomic analysis of five plant‐beneficial rhizobacterial strains ( *Acidovorax radicis* N35, *Bacillus subtilis* B171*, Bacillus velezensis* FZB42 *Rhizobium radiobacter* F4 and *Pseudomonas simiae* WCS417r), identifying shared and divergent mechanistic traits potentially linked to their positive plant effect.

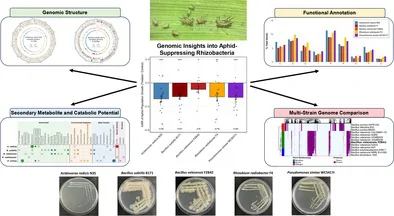

## 1 Introduction

Global demand for food is projected to increase by up to 56% by 2050 (van Dijk et al. 2021), at a time when crop productivity is increasingly constrained by human‐driven climate change. With over half of the Earth's habitable land surface currently used for agriculture, further expansion would be environmentally devastating, necessitating alternative approaches to how we farm the land (Ritchie and Roser 2019). Conventional strategies to maximise crop yields rely heavily on inorganic fertilisers and chemical pesticides. While currently effective, these strategies are associated with significant environmental damage, including the accumulation of toxic residues hazardous to humans and ecosystems (Boudh and Singh 2018; Alewu and Nosiri 2011).

The application of plant‐beneficial rhizobacteria as bioinoculants is an emerging field in sustainable agriculture, offering a sustainable alternative to chemical inputs. Microbial biofertilizers have been shown to increase crop yields by 10%–20%, depending on the climate (Schütz et al. 2018) and could reduce fertiliser application by 30% (Adesemoye et al. 2009). In addition to promoting plant growth via nutrient solubilisation, rhizobacteria can confer protection to plants against pests and pathogens via modulation of plant defences (Kumari et al. 2024). A recent meta‐analysis showed that rhizobacteria inoculation of plants can reduce leaf consumption by chewing insects and reproductive output of sucking insects, across several insect and plant families (Zytynska et al. 2025). Most included studies in the meta‐analysis examined effects following plant inoculation with *Bacillus* spp. or *Pseudomonas* spp., yet several other phylogenetically distinct rhizobacteria were found to confer insect suppression of similar magnitude. Among studies of sap‐sucking insects, aphids were by far the most frequently investigated group. Aphids are major global crop pests, siphoning plant nutrients from the phloem as they feed and transmitting economically important plant viruses (Luo et al. 2021).

Plants deploy a diverse arsenal of constitutive and inducible defences against aphids, coordinated through interconnected immune pathways and phytohormonal signalling networks, largely mediated by salicylic acid (SA), jasmonic acid (JA), and ethylene (ET) signalling (Zust and Agrawal 2016). Constitutive defences include the accumulation of secondary metabolites such as alkaloids, phenolics, and cyanogenic compounds, which directly impair aphid feeding, survival, and fecundity. Plant beneficial rhizobacteria commonly activate plant‐induced resistance (ISR) through a diverse array of bacterial‐derived signals, including volatile organic compounds, quorum‐sensing molecules, and iron‐chelating compounds, priming JA/ET‐regulated defences for more rapid and robust activation upon subsequent aphid attack (Annapurna et al. 2012; Bashir et al. 2025; Kaleh et al. 2025; del Carmen Orozco‐Mosqueda et al. 2023). In addition to modulation of plant phytohormone‐linked defences, rhizobacteria also enhance plant health through nitrogen fixation, siderophore‐mediated iron acquisition, and biocontrol of pathogens in the wider rhizosphere (Paul and Lade 2014). The beneficial effects of rhizobacteria such as *Bacillus subtilis* (Blake et al. 2021), *Pseudomonas simiae* (Pieterse et al. 2021), and *Bacillus velezensis* (Fan et al. 2018) on plant growth promotion and immune modulation, particularly against pathogens, are well established. However, in the context of rhizobacterial‐mediated aphid suppression, the array of bacterial mechanisms employed remains relatively understudied.

Successful aphid‐suppressive rhizobacteria must not only induce plant defences but also either colonise the rhizosphere or trigger persistent changes in plant responses or in the rhizosphere microbial community. Plants actively shape their rhizosphere through the secretion of root exudates, which can include primary metabolites like carbohydrates, amino acids and organic acids, or secondary metabolites such as flavonoids, auxins and glucosinolates (Vives‐Peris et al. 2020; Qu et al. 2020). Root exudates provide a substantial nutrient source for desired microbes, enhancing their establishment in the rhizosphere (Jones et al. 2009). Notably, plants can modify their exudate profiles in response to herbivory, effectively issuing a “cry for help” that selects for beneficial microbes capable of mitigating pest stresses (Rolfe et al. 2019). However, the rhizosphere is a highly competitive and dynamic microbiome, characterised by intense microbial interactions and fluctuating abiotic conditions such as nutrient availability and salinity (Jain et al. 2020). To persist within this niche, rhizobacterial strains must possess traits that boost their resilience and competitiveness, including tolerance to oxidative stress, antibiotic production, biofilm formation, and the ability to evade or modulate plant immune responses (Santoyo et al. 2021).

In this study, we conducted a comparative genomic analysis of five aphid‐suppressing rhizobacterial strains ( *Acidovorax radicis* N35, *Bacillus subtilis* B171, *Bacillus velezensis* FZB42, *Rhizobium radiobacter* F4, and *Pseudomonas simiae* WCS417r) to identify traits potentially associated with rhizobacteria‐mediated aphid suppression. All strains have been shown to reduce populations of the English grain aphid ( *Sitobion avenae* ) following root inoculation in barley ( *Hordeum vulgare* ) (Mbaluto and Zytynska 2025; Pieterse et al. 2020; Klink 2025), with suppression by *B. velezensis* further supported by unpublished data presented here. By using phylogenetically diverse strains that converge on a similar plant defence phenotype, this system provides a powerful framework to disentangle conserved and strain‐specific genomic features underpinning induced aphid resistance. In a secondary analysis, we compared each strain with closely related plant‐beneficial and pathogenic strains to place candidate traits within a broader evolutionary and functional context. Although genomic comparisons between plant‐beneficial and pathogenic bacteria have previously been used to identify traits associated with plant health (Cesbron et al. 2015; Levy et al. 2017; Siani et al. 2021), our comparative analysis instead novelly targets aphid‐suppression‐associated traits, using related strains to contextualise patterns observed in our five suppressive strains. Together, these complementary approaches allow us to assess shared or strain‐specific putative plant‐protective traits, thereby refining our understanding of the genomic basis of rhizobacteria‐mediated aphid suppression and informing the identification of functionally relevant bioinoculant traits.

## 2 Methods

### 2.1 Rhizobacterial Strains Background

*Acidovorax radicis* N35 was initially isolated from the rhizosphere of wheat ( *Triticum aestivum* ) collected in Newmarket, Germany (49.17°N, 11.28°E) in 2011 (Li et al. 2011). *Bacillus subtilis* B171 was isolated from the rhizosphere of barley ( *Hordeum vulgare* ) collected in Scheyern, Germany (48.29°N, 11.26°E) in 2004 (Schreiner 2008). *Bacillus velezensis* FZB42 was isolated from the rhizosphere of beet ( *Beta vulgaris* ) collected in Göttingen, Germany (51.54°N, 9.92°E) in 1997 (Krebs et al. 1998). *Pseudomonas simiae* WCS417r was isolated from the rhizosphere of wheat ( *Triticum aestivum* ) collected in Flevopolder, the Netherlands (52.50°N, 5.47°E) in 1988 (Lamers et al. 1988). *Rhizobium radiobacter* F4 was isolated as an endofungal bacterium from *Piriformospora indica* collected in the Thar Desert, India (27.47°N, 70.62° E) in 2008 (Sharma et al. 2008).

### 2.2 Aphid Suppression Experiments

The dataset for the aphid suppression analysis has been collated from three separate unpublished experiments using the five rhizobacteria strains (1) 2019: *R. radiobacter* F4 (*n* = 16) (barley varieties Barke, Chevallier, Scarlett); (2) 2020: *A. radicis* N35 (*n* = 9), *Bacillus subtilis* B171 (*n* = 9), *B. velezensis* FZB42 (*n* = 9), and *P. simiae* WCS417r (*n* = 9) (barley variety Irina); (3) 2021: *A. radicis* (*n* = 11), *B* *. subtilis* (*n* = 12), *P. simiae* (*n* = 11) (barley variety Irina). Each followed the same experimental protocol. Bacterial strains were each grown on nutrient agar plates [Difco General purpose Nutrient Broth 8 g/L plus 15 g agar] at 30°C for 5 days and then harvested and suspended in 10 mM MgCl_2_ at OD_600_ = 2.0. Barley seeds ( *Hordeum vulgare* ) were surface sterilised in bleach and germinated in between moist filter paper in petri dishes for 5 days. Seedlings were inoculated with rhizobacteria (or a control solution of MgCl_2_) by soaking in bacterial suspension for 2 h and planted into individual pots containing low‐nutrient soil substrate. Two 4th instar aphids ( *Sitobion avenae* ) were introduced to plants once they reached a shoot length of 10 cm, plants were covered in fine mesh, and the total number of aphids was counted after 14 days. Plants were all grown under controlled conditions (18°C–20°C, 60%–65% RH, 16:8 L:D). Negative binomial models were used to analyse the effect of rhizobacteria inoculation on aphid number after 2 weeks of growth, including experiment and barley variety as random effects, with the control group as the focal contrast for post hoc analyses.

### 2.3 Rhizobacterial Genome Sequencing and Annotation

*Acidovorax radicis* N35, *Bacillus subtilis* B171, *Bacillus velezensis* FZB42, *Rhizobium radiobacter* F4 and *Pseudomonas simiae* WCS417r were cultured in Nutrient Broth (8 g/L) at 30°C for 48 h. Whole‐genome sequencing was performed by MicrobeNG (Birmingham, UK) using Oxford Nanopore Technologies long‐read sequencing (R10.4.1 chemistry). Assembled genomes were deposited in the GenBank NIH genetic sequence database (PRJNA1433263). Plasmids were identified using PlasmidHunter (v1.1) (Tian et al. 2024), with *R. radiobacter* plasmid identities confirmed via literature review (Glaeser et al. 2016). Phage sequences were predicted using PHASTER (Arndt et al. 2016). Functional annotation was performed using EggNOG‐mapper (v2) (Cantalapiedra et al. 2021) against the EggNOG 5.0 database (Huerta‐Cepas et al. 2019). Gene counts for each COG functional category were normalised to the total genome and expressed as percentages. Poisson generalised linear models were fitted for each COG functional category to test genome effects on gene counts, assessed using likelihood ratio chi‐square tests. Pairwise post hoc contrasts between genomes were conducted using estimated marginal means. Circular genome plots were generated using Bakta (v1.11) (Schwengers et al. 2021). Secondary metabolite clusters were predicted with AntiSMASH (v8.0) (Blin et al. 2025) and clustered by similarity using Clinker (minimum alignment identity ≥ 0.5) (Gilchrist and Chooi 2021). Catabolic potential was assessed using RhizoSMASH (v0.3.1) (Li et al. 2025), and barley exudates were identified via a review of the literature (Calvo et al. 2019; Naveed et al. 2017; Shaposhnikov et al. 2024; Pacheco‐Moreno et al. 2024). Genes associated with beneficial plant interaction were predicted using PLaBase's PGPT‐Pred service (Patz et al. 2021).

### 2.4 Genomic Comparison of Closely Related Plant‐Beneficial and Pathogenic Rhizobacteria

A total of 51 strains (Table S1) were downloaded from the NCBI genome database (accessed 24.09.25) by searching for genomes closely related to *Acidovorax radicis* N35, *Bacillus subtilis* B171, *Bacillus velezensis* FZB42, *Rhizobium radiobacter* F4, and *Pseudomonas simiae* WCS417r. Literature was then reviewed to identify strains with reported direct plant interactions, selecting at least nine strains for each focal strain to represent different types of plant associations (see Table S1). Strains were classified as ‘plant beneficial’ if published data demonstrated positive effects on plant health, including plant growth promotion or enhanced pest suppression. Strains were classified as ‘plant neutral’ if studies reported no measurable effects on plant health or if no information on plant interactions was available. Strains were classified as ‘plant pathogenic/phytopathogenic’ if published data demonstrated pathogenic interactions with plants. For the *Bacillus* subgroups, strains exhibiting context‐dependent pathogenicity or genomic traits linked with pathogenicity were also included in this category because of the limited number of recognised plant pathogens within this genus.

Protein sequences were predicted using Prokka (v1.14.6) (Seemann 2014). Phylogenetic relationships were inferred with PhyloPhlAn (v3.1.68) (Asnicar et al. 2020) using the high diversity setting and default universal marker database of 400 genes. Branch support was assessed with RAxML (v8.2.13) (Stamatakis 2014), and the tree was visualised using ITOL (v7.4.1) (Letunic and Bork 2024). Non‐core genes shared by all beneficial or pathogenic strains within each related strain subgroup were identified using Roary (v3.13.0) (Page et al. 2015), excluding genes present in both subgroups. Gene order was conserved across related strain sets to enable comparison of homologous genes among taxa. FastANI (v1.34) (Jain et al. 2018) was used to assess Average Nucleotide Identity. Functional annotation was performed using KofamScan (KEGG database release 116.0, version 2025‐12‐01) (Aramaki et al. 2020). Plant‐beneficial and virulence‐associated genes were predicted using PLaBase PGPT‐Pred (v1.02) (Patz et al. 2024, 2021; Martinez‐Garcia et al. 2016) and PIFAR‐Pred (v1.02) (Patz et al. 2021, 2024; Ashrafi et al. 2022) services (Patz et al. 2021).

### 2.5 Figure Generation and Statistical Testing

Data visualisation and statistical analyses were performed using R version 4.4.2 in RStudio 2025.09.1. Data manipulation was performed using Tidyverse packages dplyr (v1.1.4), tidyr (v1.3.2), purr (v1.2.1). Figures were generated using ggplot2 (v4.0.1), with colour palettes from RColorBrewer (v1.1–3) and scaling via the Scales (v1.4.0) packages. The presence/absence map was produced using ComplexHeatmap (v2.26.0) and Circlize (0.4.17).

Poisson generalised linear models were used for genome‐level gene count data, and negative binomial mixed‐effects models were fitted for aphid count data using the GlmmTMB (v1.1.14) package. Model assumptions were evaluated using DHARMa (0.4.7).

## 3 Results

### 3.1 Aphid Suppression

**FIGURE 1:**
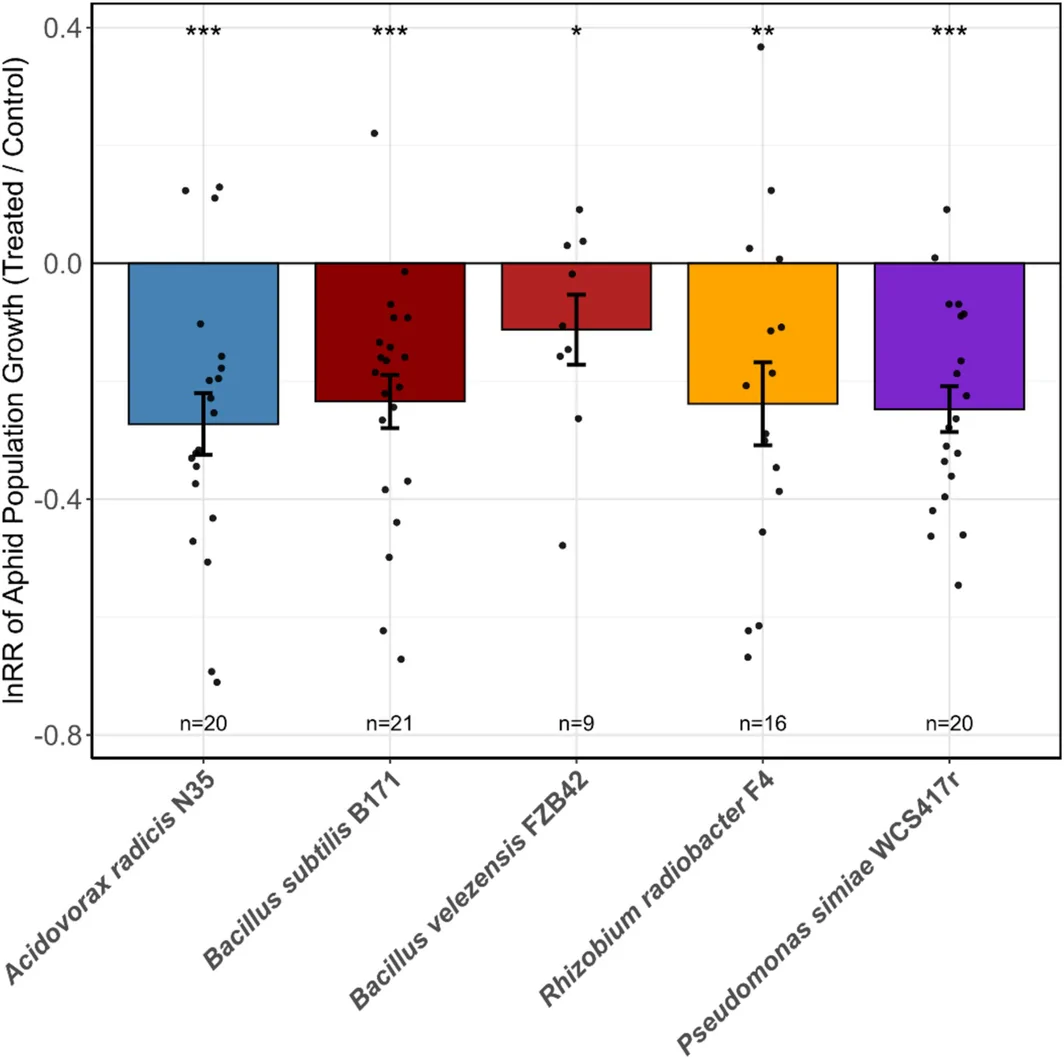
Aphid population growth on barley plants after rhizobacterial inoculation compared with control/inoculated plants (Log Response Ratio) across multiple experiments. Error bars show mean ± SE, with individual data points also displayed. Sample size for each strain is given below the bars (*n* = 20, 21, 9, 16, 20). Asterisks indicate significance: *p < 0.05, **p < 0.01, ***p < 0.001.

To estimate the impact of the five divergent rhizobacteria on aphid population growth on barley plants, we grew plants with and without inoculation with each pest‐suppressing rhizobacterium. We detected a significant effect of each focal rhizobacterium (Χ^2^ = 27.07, df = 5, *p* < 0.001): *Acidovorax radicis* N35 (*z* = −3.51, *p* < 0.001), *Bacillus subtilis* B171 (*z* = −3.13, *p* = 0.002), *Bacillus velezensis* FZB42 (*z* = −2.20, *p* = 0.028), *Rhizobium radiobacter* F4 (*z* = −3.26, *p* = 0.001), and *Pseudomonas simiae* WCS417r (*z* = −3.36, *p* < 0.001) (Figure 1), with each inoculant reducing aphid load by ~25%, apart from *B. velezensis* , that displays a 10% reduction.

### 3.2 Genomic Characteristics

Next, we sequenced, assembled, and compared the genomes of the different focal strains to identify genus differences and common features that might explain the aphid suppression trait. Despite their shared plant impact, the genomic profiles of the focal strains possess both divergent and shared characteristics (Table 1). The two *Bacillus* strains, *B. subtilis* and *B. velezensis* , have smaller genome sizes and lower numbers of genes compared with *A. radicis* , *P. simiae* , and *R. radiobacter* , consistent with established taxonomic variation (Wu et al. 2025). *Rhizobium radiobacter* is distinct due to its genome structure, possessing three replicons: a circular chromosome and plasmid, and a putative linear chromid (Kuzmanovic et al. 2025).

**TABLE 1:** An overview of the genomic characteristics of the five rhizobacterial strains.

Strain	Taxonomy	Morphology	Genome size (bps)	GC (%)	Genes (cds)	Plasmids	Phages^a^	Plate morphology
*Acidovorax radicis* N35	Pseudomonadota; Betaproteobacteria; Burkholderiales; Comamonadaceae; Acidovorax	Gram Negative Rod	5,542,670	64.8	5024	0	1 intact prophage: 27 kps (PHAGE_Burkho_KS14)	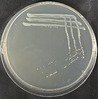
*Bacillus subtilis* B171	Bacillota; Bacilli; Bacillales; Bacillaceae; Bacillus	Gram Positive Rod	4,286,715	43.5	4317	0	2 intact prophages: 33.7 kps (PHAGE_Brevib_Jimmer2), 127.1 kps (PHAGE_Bacill_SPbeta)	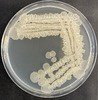
*Bacillus velezensis* FZB42	Bacillota; Bacilli; Bacillales; Bacillaceae; Bacillus	Gram Positive Rod	3,915,549	46.5	3728	0	0 intact prophages	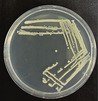
*Rhizobium radiobacter* F4	Pseudomonadota; Alphaproteobacteria; Hyphomicrobiales; Rhizobiaceae; Rhizobium	Gram Negative Rod	5,649,947 Total (Circular: 2,811,183) Linear: 2,066,479 Plasmid: 772,285	59.1	5255	2 Plasmids: pTiF4, ~0.55 mb pAtF4, ~0.22 mb	1 intact prophage: 12.2 kps (PHAGE_Geobac_GBK2)	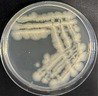
*Pseudomonas simiae* WCS417r	Pseudomonadota; Gammaproteobacteria; Pseudomonadales; Pseudomonadaceae; Pseudomonas	Gram Negative Rod	6,185,137	60.3	5591	0	2 intact prophages: 20.4 kps (PHAGE_Vibrio_VP882), 53.6 kps (PHAGE_Pseudo_YMC11/02/R656)	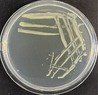

*Note:* The first column is colored by bacterial strain with the strain colour scheme maintained throughout.

^a^ Phages detected via Phaster (Arndt et al. 2016).

**FIGURE 2:**
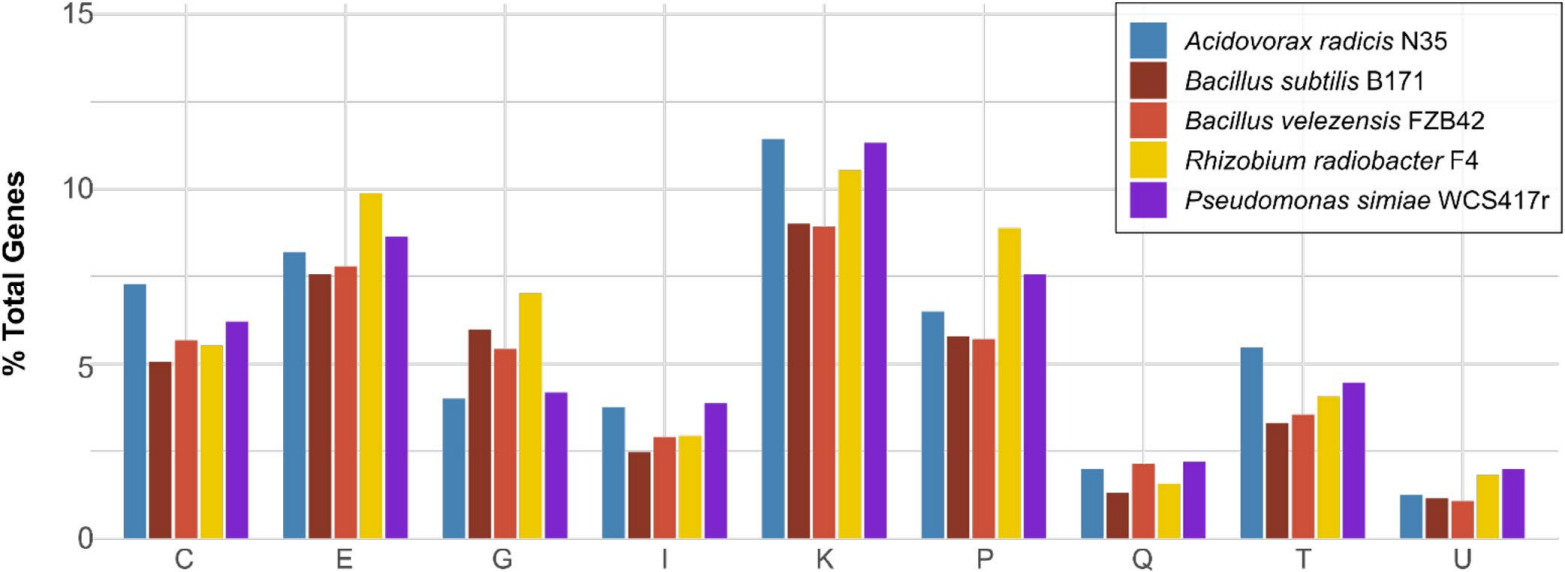
Comparison of COG functional categories across strains. Only categories showing significant differences (*p* < 0.05 Benjamini‐Hochberg adjusted) after χ^2^ tests are displayed. (C = Energy production and conversion, E = Amino acid transport and metabolism, G = Carbohydrate transport and metabolism, I = Lipid transport and metabolism, K = Transcription, *P* = Inorganic ion transport and metabolism, Q = Secondary metabolites biosynthesis, transport and catabolism, *R* = General function prediction only, T = Signal transduction mechanisms, U = Intracellular trafficking, secretion, and vesicular transport, W = Extracellular structures, X = Mobilome: Prophages, transposons, Y = Nuclear structure, Z = Cytoskeleton).

Examining genus differences across functional categories reveals that carbohydrate transport and metabolism genes (Figure 2 category G) differ significantly among the focal strains (χ^2^ = 61.19, df = 4, *p* < 0.001), with *A. radicis* and *P. simiae* encoding fewer carbohydrate transport and metabolism genes than the other strains. *Rhizobium radiobacter* encodes significantly more genes for inorganic ion transport and metabolism (χ^2^ = 119.70, df = 4, *p* < 0.001; *R. radiobacter* post hoc *z* = 4.8, *p* < 0.001; Figure 2, category P) and *A. radicis* for signal transduction mechanisms (χ^2^ = 65.76, df = 4, *p* < 0.001, *A. radicis* post hoc *z* = 2.3, *p* < 0.05; Figure 2, category T). The two closest related *Bacillus* strains have very similar gene counts in most categories; however, they exhibit variation in the number of secondary metabolite‐associated genes ( *B. subtilis* *n* = 51, *B. velezensis* *n* = 84; Figure 2, category Q).

**FIGURE 3:**
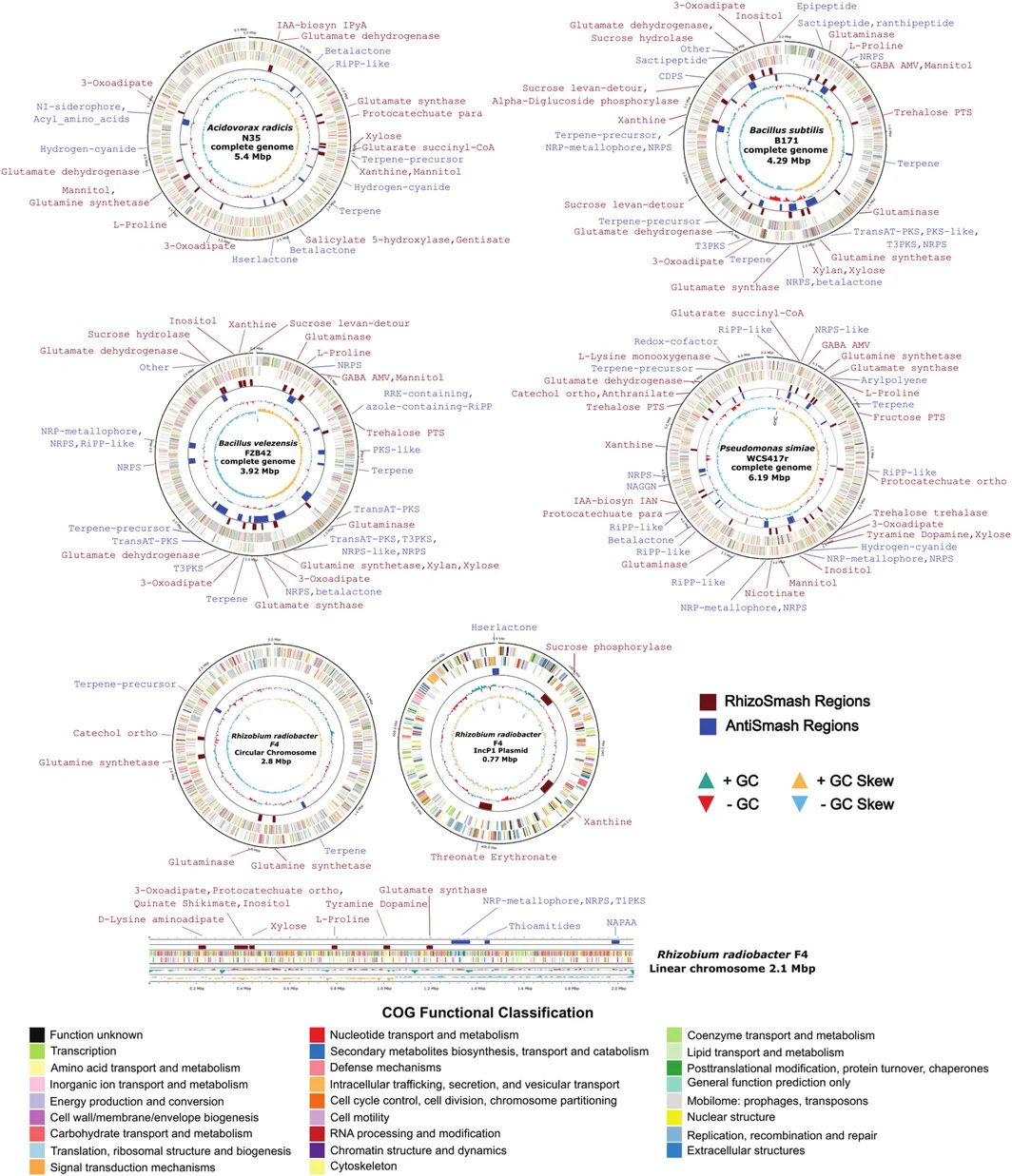
Genome visualisation of the five focal strains, plotted using Bakta (Schwengers et al. 2021). COG annotation has been represented in the outer ring for the forward strand and the second inner ring for the reverse strand. Also highlighted are gene clusters identified by RhizoSMASH (Li et al. 2025) (indicating the strain's catabolic potential within the rhizosphere) and AntiSMASH (Blin et al. 2025) (predicting the strain's secondary metabolite profile).

Next, we investigated genomic regions predicted to be involved in secondary metabolite production to evaluate each strain's competitive profile and identify metabolites potentially involved in aphid suppression. Annotating AntiSMASH (Blin et al. 2025) regions on genome plots (Figure 3) revealed variation in the number of secondary metabolite biosynthetic regions each focal strain possesses. *P. simiae* has the most regions (*n* = 16), then the two *Bacillus* species (*n* = 13), followed by *A. radicis* (*n* = 9), and finally *R. radiobacter* has the least (*n* = 6). At a high level, only terpenes are produced by all focal strains, with variation in every other type of metabolite. RhizoSMASH (Li et al. 2025) analysis (Figure 3) revealed that every strain possesses catabolic regions linked to degrading glutamine/glutamate, xylose and xanthine, among others, but only *A. radicis* and *P. simiae* have regions associated with degrading plant phytohormones (Figure 3).

### 3.3 Secondary Metabolite and Catabolic Potential

**FIGURE 4:**
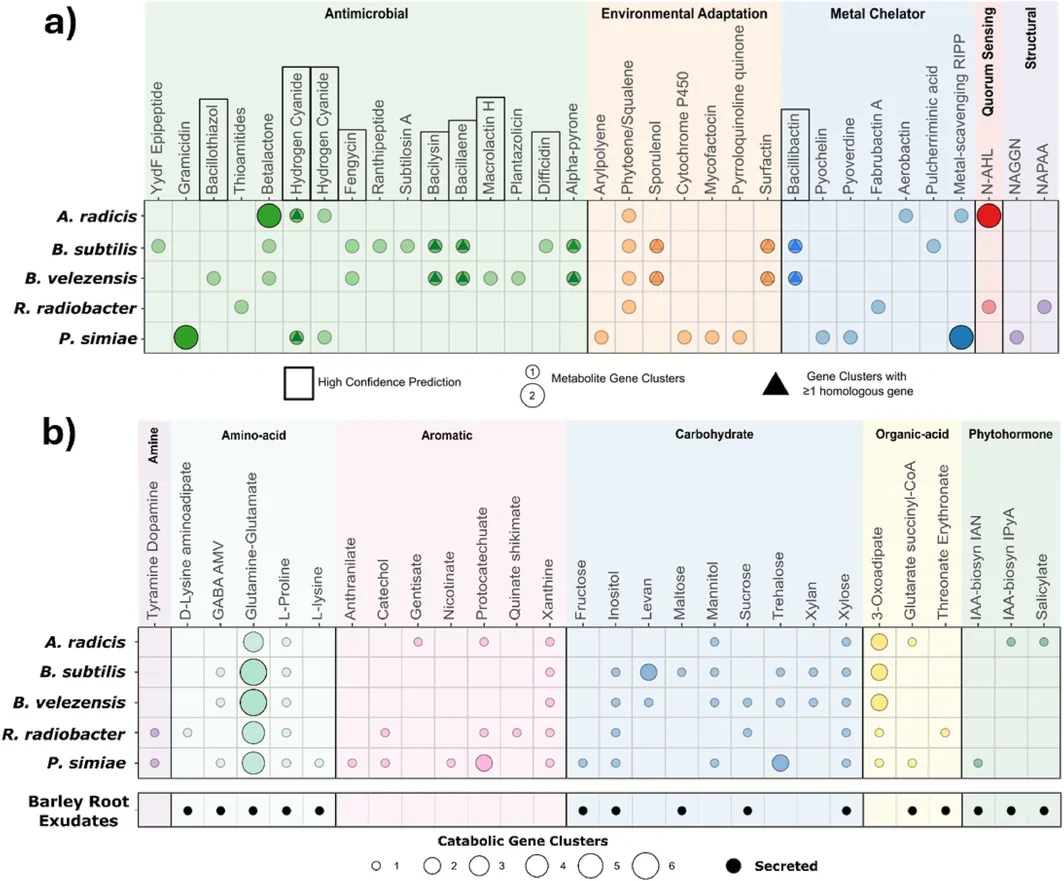
Metabolic and catabolic potential of the five focal strains (a) Secondary metabolite potential of each focal strain predicted using AntiSMASH (Blin et al. 2025). Metabolites are grouped by their primary role in soil, although they may possess multiple functions. High‐confidence predictions (boxed) have a strong similarity to known metabolites, while others have been inferred from gene function. (b) Catabolic potential predicted using RhizoSMASH (Li et al. 2025), showing substrate degradation capacity in the rhizosphere. Substrates are categorised by structure/function, and those secreted by barley through root exudation are highlighted. Across both figures, the point size indicates the number of gene clusters per strain.

To further explore the secondary metabolite potential, we predicted the identity of metabolites produced by the focal strains and categorised them into functional groups relevant to the rhizosphere (Figure 4a). The two *Bacillus* strains share a similar metabolite profile (Figure 4a) but differ in their production of antimicrobial metabolites. Both *A. radicis* and *P. simiae* produce hydrogen cyanide, an antimicrobial also linked to plant signalling and herbivory defences in plants (Diaz‐Rueda et al. 2023). These two strains also encode multiple copies of biosynthetic gene clusters across the different groups, indicating redundancy or potential for increased production of these metabolites. All except *P. simiae* can produce phytoene/squalene (multi‐functional synthase gene cluster), linked to antioxidant activity (Zhang et al. 2025; Xu et al. 2016). *Pseudomonas simiae* also produces metabolites linked to antioxidants: aryl polyene, mycofactocin and pyrroloquinoline quinone (Schoner et al. 2016; Ellerhorst et al. 2025; Misra et al. 2012). In addition, every strain engages in metal chelation, all producing a type of siderophore (a common function in the rhizosphere), but specific to the bacterial genus (Figure 4a). Predictions for *R. radiobacter* metabolites are fewer, with reduced diversity and gene clusters compared with the other strains. However, *R. radiobacter* does produce at least one metabolite across all functional categories, including the uncommon N‐AHL‐based quorum‐sensing category, which was otherwise only detected in *A. radicis* .

Using RhizoSMASH's (Li et al. 2025) predictions of the catabolic potential of the strains, we identified common substrates that all strains can degrade; these include glutamine‐glutamate, L‐proline, xanthine, xylose, and 3‐oxoadipate (Figure 4b). Our experimental system uses barley (Figure 1), whose root exudate profile contains all these substrates except xanthine and 3‐oxoadipate (Figure 4b). Glutamine‐glutamate, L‐proline, and xylose production in plants have all been shown to shift in response to herbivory and other stresses (Blin et al. 2025; Steinbrenner et al. 2011; Cornara et al. 2023; Verslues and Sharma 2010; Zoric et al. 2019). The strains also catabolise additional substrates released by plants into the rhizosphere, with variation in substrate range (Figure 4b). Interestingly, only *A. radicis* and *P. simiae* have the potential to catabolise phytohormones, either as a step in their own production of these compounds or targeting those released by plants (Figure 4b).

### 3.4 Linking Microbial Function to Plant Immunity

**FIGURE 5:**
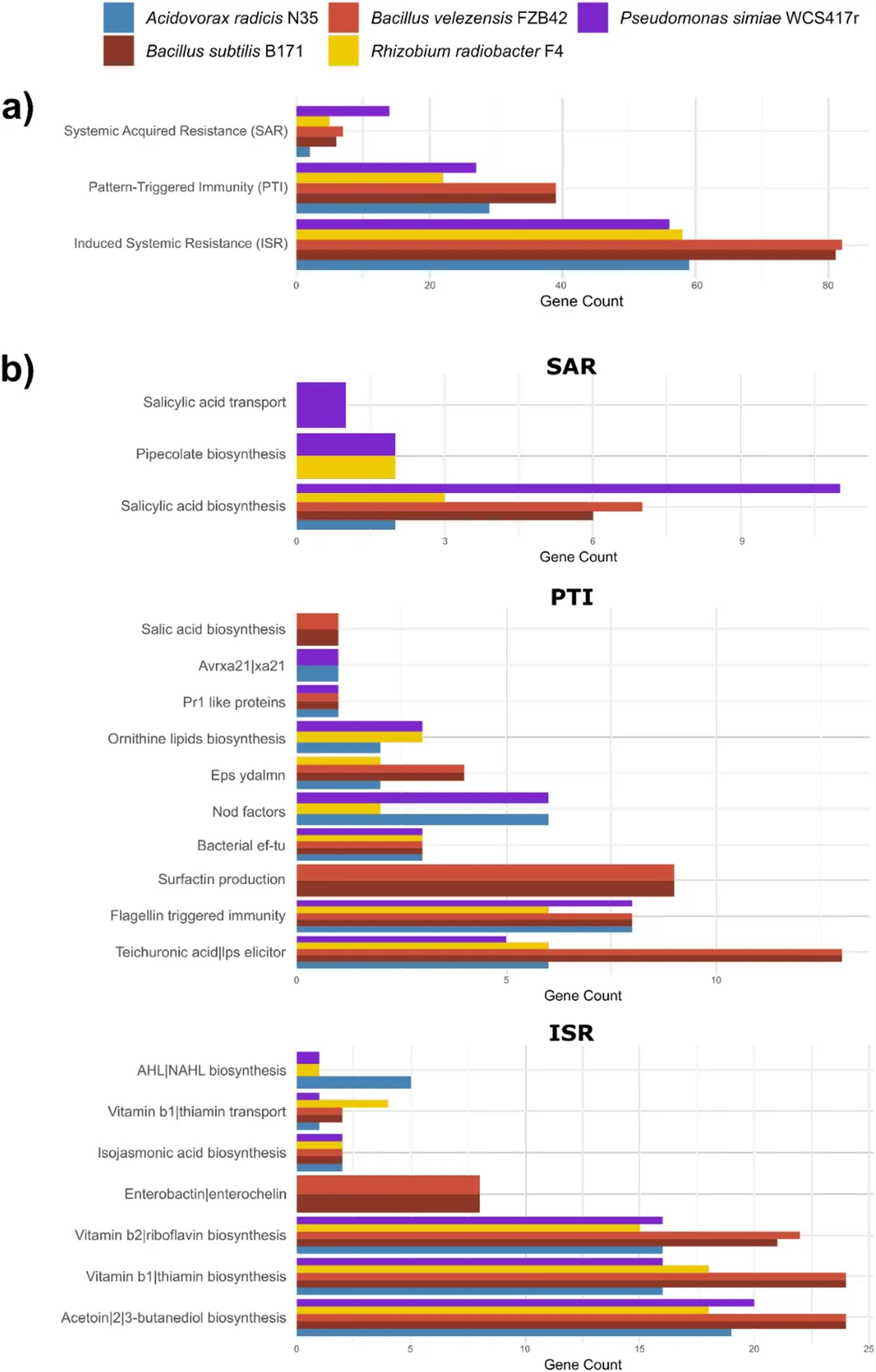
Bacterial genes linked to plant immune system activation in each focal strain. (a) Focal strain genes associated with triggering three high‐level plant immune defence pathways (Systemic acquired resistance, Pattern‐triggered immunity and Induced systemic resistance) (b) Focal strain genes separated into specific mechanisms associated with triggering each plant immune defence pathway. Gene links to plant immunity were identified using PlaBase (Patz et al. 2021).

To discover the potential of each strain to modulate plant immune defences, we analysed their genomes using PLaBase (PGPT‐Pred) (Ashrafi et al. 2022; Patz et al. 2021, 2024) (Figure 5). When genes were grouped into major plant immunity pathways, induced systemic resistance was associated with the greatest number of genes across all strains, followed by pattern‐triggered immunity, while systemic acquired resistance had the fewest. The two *Bacillus* species possess more genes linked to pattern‐triggered immunity and induced systemic resistance than the others, while *P. simiae* has more genes linked with systemic acquired resistance compared with the rest (Figure 5a). Breaking down the three modulated plant immune pathways into specific functions (Figure 5b), we observe common mechanisms among all focal strains with comparable gene counts. Riboflavin, thiamine and butanediol are synthesised by every focal strain, and these three pathways have the highest number of associated genes (Figure 5b), although this may not correlate directly with production. There are other common pathways, such as LPS elicitor and salicylic acid biosynthesis, but the gene counts differ depending on the strain (Figure 5b). There are divergent pathways among the strains as well, with only the *Bacillus* strains producing surfactin, and only the non‐*Bacillus* strains encoding *N*‐AHL biosynthesis genes, with *A. radicis* possessing more than *P. simiae* and *R. radiobacter* (Figure 5b). This differs from the AntiSMASH (Blin et al. 2025) analysis (Figure 4a), where no N‐AHL biosynthetic gene clusters were detected for *P. simiae* . There is only one pathway that is unique to a single strain, salicylic acid transport, with only *R. radiobacter* possessing two associated genes.

### 3.5 Comparing Plant Beneficial Strains With Related Pathogenic Counterparts

**FIGURE 6:**
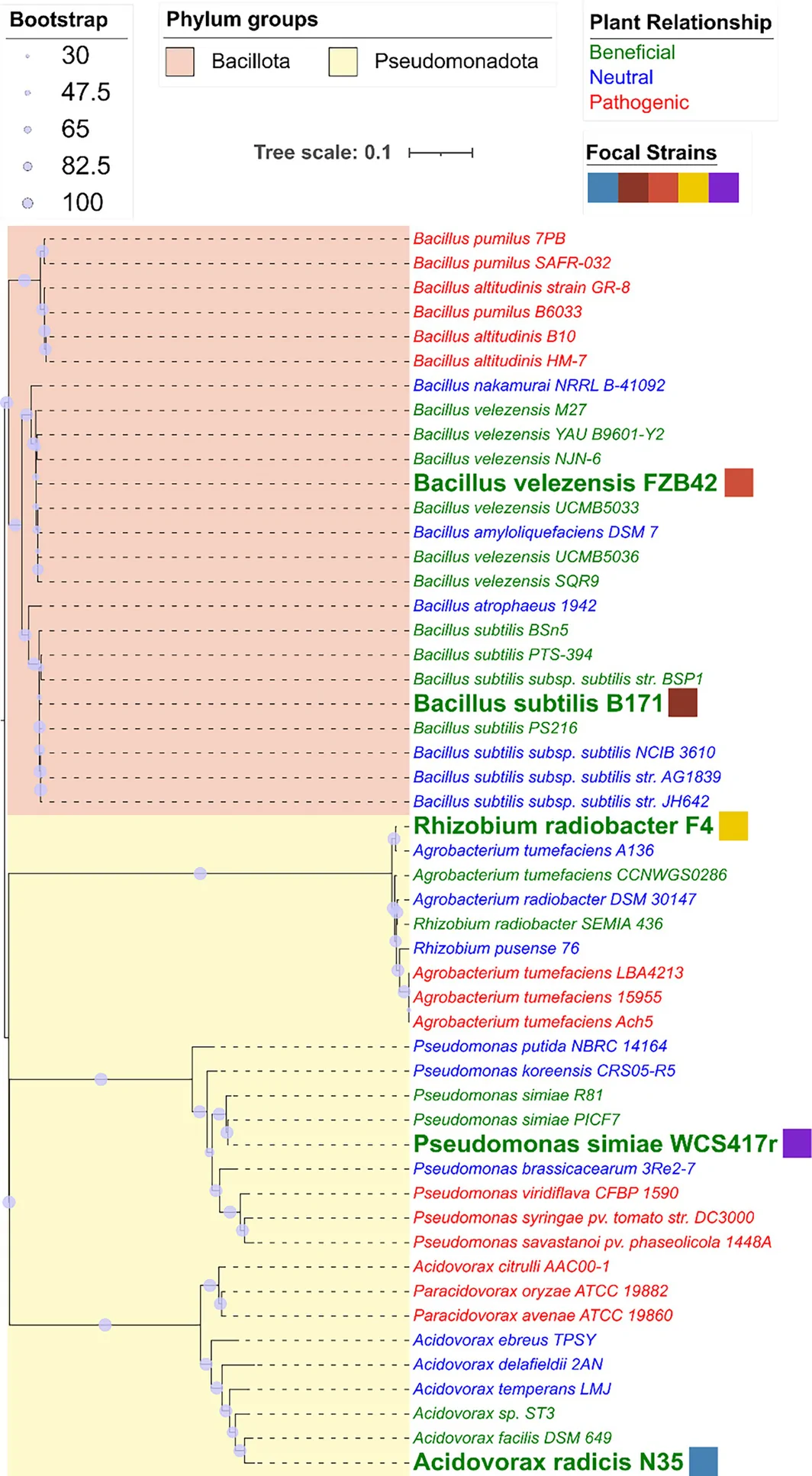
Phylogenetic tree of the five focal rhizobacterial strains (highlighted), aligned with related plant beneficial, neutral, or pathogenic strains, established from published studies. All strains were originally isolated from soil: Beneficial strains (green), neutral strains (blue) and pathogenic strains (red). Alignments were generated using 400 marker genes from Phylophlan's universal marker database.

To identify potential functional patterns between plant‐beneficial and phytopathogenic strains, a set of related genomes was extracted from the NCBI database for each focal strain, encompassing plant‐beneficial, neutral, and pathogenic strains (Figure 6). Beneficial and phytopathogenic strains cluster separately in each set, although the phylogenetic distance for the *R. radiobacter* groups is notably smaller than that observed for the other focal strains.

**FIGURE 7:**
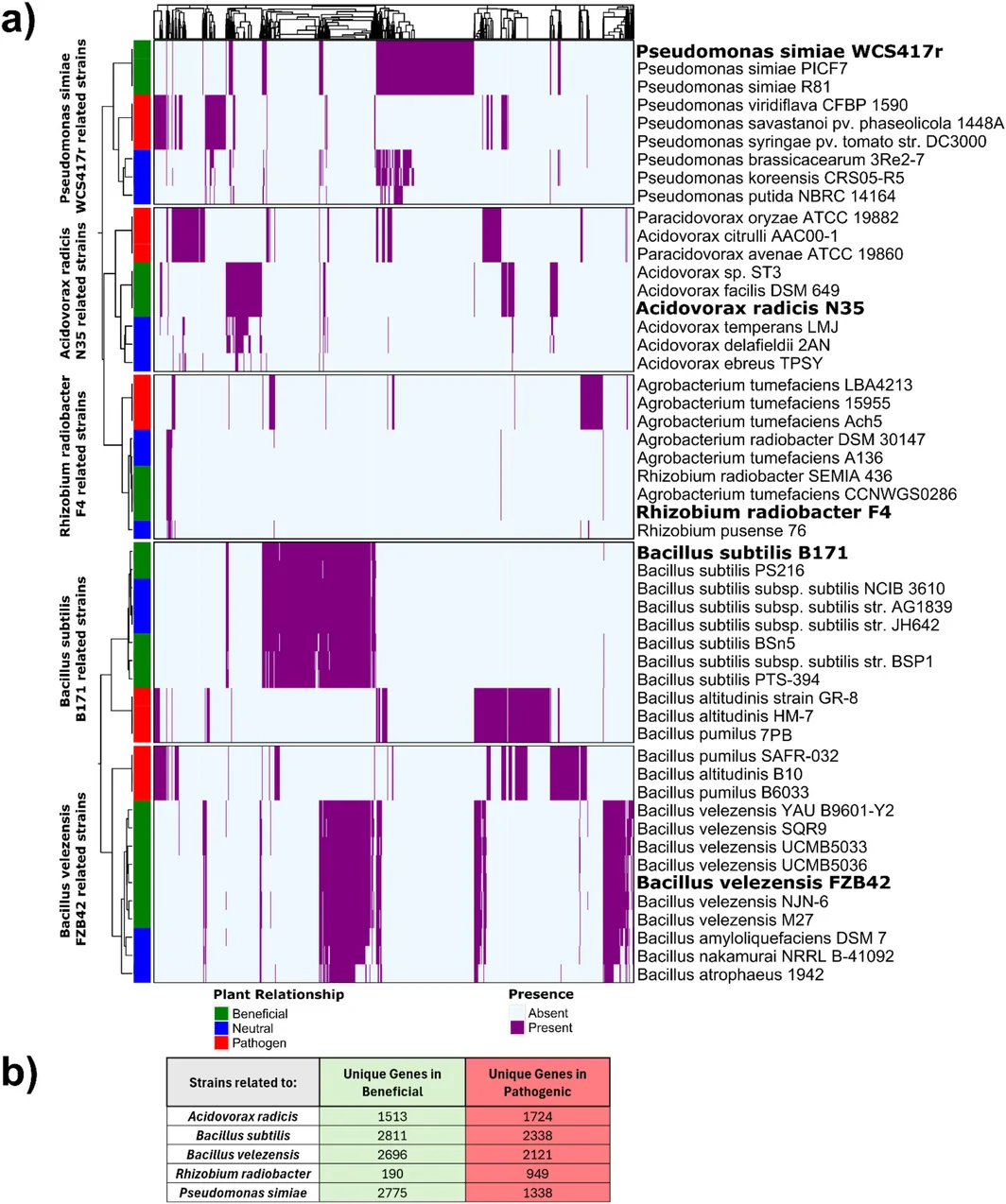
Gene presence and count across focal and related strains (a) non‐core genes unique to plant‐beneficial or pathogenic strain subgroups. Neutral strains are displayed for comparison, but no filtering was performed due to their unconfirmed plant relationship. Gene order is maintained across subgroups, allowing comparison (b) unique gene counts for each group.

Within each focal strain set, plant beneficial and phytopathogenic genomes were compared using the pan‐genome package Roary (Page et al. 2015) (Figure 7). Non‐core genes were identified that were present in every beneficial and phytopathogenic strain, and genes common to both were excluded to obtain unique subgroup‐specific sets (Figure 7a). The *Bacillus* strains display many conserved genes for their beneficial subgroups, shown by large gene blocks in the same position on both maps, implying similar plant beneficial functions in each *Bacillus* set. To determine whether these shared regions reflected phylogenetic relatedness rather than convergent functional adaptation, Average Nucleotide Identity (ANI) was calculated using FastANI (Jain et al. 2018). Beneficial *Bacillus* strains shared > 80% genome similarity with each other, whereas pathogenic strains from the corresponding groups showed < 80% similarity. This pattern suggests that the conserved gene blocks among beneficial strains may largely reflect shared evolutionary ancestry.

Each focal strain subgroup has a large selection of unique genes identified (> 1000) (Figure 7b), apart from the *R. radiobacter* groups, which are low for the phytopathogenic group (*n* = 949), and even lower for the beneficial group (*n* = 190). This shows distinct variation for most focal strain sets between the related plant beneficial and phytopathogenic strains. However, *R. radiobacter* strains are an exception, with a high number of conserved genes filtered out, leaving fewer unique genes in each subgroup.

**FIGURE 8:**
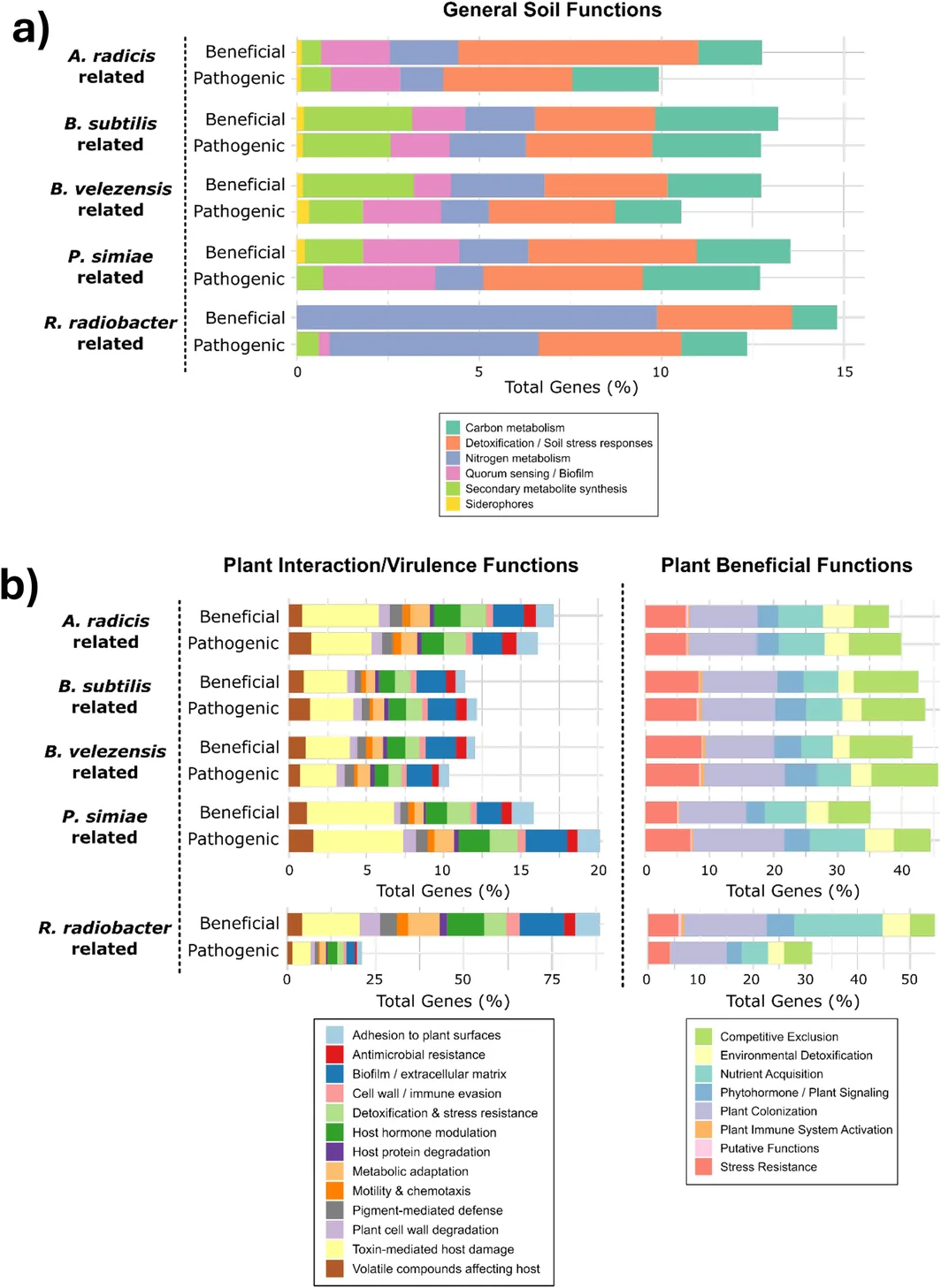
Soil and plant‐relevant functions within unique subgroup genes (a) KEGG‐based functional classification of unique genes in plant beneficial and pathogenic subgroups, filtered to those important in the rhizosphere. (b) Also analysed using PLaBase (Patz et al. 2021), predicting plant interaction/virulence and plant beneficial functions.

Functional classification of genes unique to beneficial and pathogenic subgroups reveals common and divergent features within each focal strain set (Figure 8). Phylogenetic similarity may contribute to recurring traits within related strain groups; however, the observation of consistent patterns across four taxonomically distinct groups mitigates this concern. For both beneficial and pathogenic subgroups, 10%–15% of genes are associated with general soil functions (KEGG‐classifier; Figure 8a). Common trends can be identified, with all beneficial groups possessing a similar percentage of detoxification/soil stress response‐linked genes when compared with their corresponding pathogenic groups, with the *A. radicis* set as the only exception. This set instead has a higher percentage in its beneficial subgroup compared with its pathogenic. The *B. velezensis* and *P. simiae* sets show a similar trend for other functions, with the pathogenic subgroup having a lower percentage of secondary metabolite synthesis‐linked genes than the beneficial. The *R. radiobacter* set differs, with the pathogenic subgroup possessing more genes linked to secondary metabolite synthesis and quorum sensing, while the beneficial subgroup does not have any. This is balanced by the beneficial subgroup having a much higher percentage of genes associated with nitrogen metabolism. The *B. subtilis* subgroups have a similar profile, with minor variation in secondary metabolite synthesis and carbon metabolism‐linked genes.

When classifying genes for plant‐specific functions, 7%–20% of genes are linked with interaction/virulence functions (from PLaBase (Patz et al. 2021; Patz et al. 2024; Martinez‐Garcia et al. 2016) PIFAR‐Pred; Figure 8b), and 30%–55% of genes could be linked to positive plant functions (from PLaBase (Ashrafi et al. 2022; Patz et al. 2021; Patz et al. 2024) PGPT‐Pred; Figure 8b). An exception is the *R. radiobacter* beneficial subgroup, which has > 80% of genes predicted to be linked with plant interaction/virulence. The beneficial and pathogenic subgroups for *A. radicis, B. subtilis* and *B. velezensis* ‐related strains have a similar overall profile, with only a few minor variations. One difference is in the percentage of genes linked with volatile compounds affecting the host for *A. radicis* and *B. subtilis* , along with *P. simiae* , where the pathogenic subgroups possess a higher percentage than their beneficial counterparts. The *P. simae* set also shows variation in other functions, with the pathogenic strains containing a higher percentage of genes related to biofilm/extracellular matrix, host hormone modulation, and plant cell wall degradation than the beneficial strains, but also a higher percentage of genes linked to beneficial functions such as stress resistance, plant colonisation, and nutrient acquisition. Overall, the *R. radiobacter‐*related‐strain set shows the greatest variation between its two subgroups (> 60%; Figure 8b). The beneficial group possesses a higher percentage of genes for every function, with biofilm/extracellular matrix and metabolic adaptation having the largest differences. This indicates that a high percentage of the unique genes that differentiate *R. radiobacter‐*related beneficial strains from pathogenic strains are directly linked with plant interactions.

When classifying the unique genes by potential plant beneficial functions (Figure 8b), the *A. radicis* and *B. subtilis* sets show the greatest functional similarity between their beneficial and pathogenic strains, but differences are still present. The *A. radicis* beneficial subgroup has a higher percentage of genes linked with environmental detoxification (a common plant beneficial trait), while its pathogenic group has a higher percentage of genes associated with impeding the growth of other microbes (competitive exclusion). The *B. subtilis* subgroups differ in more unexpected functions, with the pathogenic subgroup having a higher percentage of genes involved in phytohormone/plant signalling and environmental detoxification, often considered more plant beneficial functions. The *P. simiae* and *R. radiobacter* sets display a larger variation between their subgroups (Figure 8b). The *R. radiobacter* beneficial subgroups have a higher percentage of genes related to stress resistance than their respective pathogenic groups, while the *P. simiae* set shows the opposite. Nutrient acquisition‐linked genes are another example of this trend, with a higher percentage of genes in the *P. simiae* pathogenic subgroup compared with its beneficial subgroup, which is reversed for the *R. radiobacter* set. Therefore, while there are differences between plant beneficial and pathogenic strains within related strain sets, no consistent pattern emerges overall across the differing taxonomic groups.

## 4 Discussion

Our comparison of five taxonomically distinct rhizobacterial strains (focal strains), each capable of inducing defence responses in barley against aphid pests, revealed substantial functional variation. Several functions shared across multiple strains were identified that are predicted to provide plant benefits and provide potential candidates for inducing aphid suppression. These include a dual phytoene/squalene biosynthetic gene cluster, the biosynthesis of 2,3‐butanediol, riboflavin, thiamin, and salicylic acid, along with quorum‐sensing capabilities, all predicted to induce plant defences. Other functions of interest include the well‐known *Bacillus* strains' broad antimicrobial profiles, as well as *A. radicis* and *P. simiae's* synthesis of hydrogen cyanide and interaction with plant phytohormone IAA.

That the focal strains exhibit large variation in their functional profile is not unexpected due to the phylogenetic relatedness of these strains. However, our aim was to examine these profiles to identify common candidate traits linked to aphid suppression that are shared despite the taxonomic distance. The strains are also soil‐dwelling, with no direct interaction with aphids on plant leaves, so the traits identified are either indirectly beneficial or linked with the already established bacterial induction of the plant's immune defences (Borah et al. 2023).

One trait that is conserved across focal strains, apart from *P. simiae*, is the encoding of a dual phytoene/squalene biosynthetic gene cluster. These terpenes primarily act as antioxidants in the rhizosphere, enabling bacteria to cope with oxidative stress, but they are also intermediaries in other pathways (Xu et al. 2007; Ghimire et al. 2016). Notably, these include the carotenoids and hopanoids pathways, which have also been shown to act as plant signalling molecules (Vriet et al. 2013; Cazzonelli 2011). Studies do not yet demonstrate the direct link between bacterially synthesised variants of these compounds and plant signalling, but exogenously applied carotenoids result in increased plant growth (Shen et al. 2019). Disrupting hopanoid production in strains within root nodules also reduced their effect on plant immune defences (Belin et al. 2018). This suggests that microbially‐derived phytoene/squalene terpenes can modulate plant processes and potentially induce plant pest defences.

All focal strains were predicted to interact with the plant immune system, with our analyses revealing multiple aphid‐suppressing candidate traits. Bacterial synthesis of salicylic acid (SA) was a shared function across all strains, a key plant phytohormone leading to activation of plant immune defences, including certain aphid resistance mechanisms. Exogenously applied SA triggers activation of SA‐dependent defence pathways without requiring transcriptional activation within the plant, lowering the metabolic cost of this defence pathway (Chen et al. 2025). However, it is uncertain whether microbe‐derived SA is directly released into the rhizosphere, instead being incorporated into other compounds that may trigger plant immune defences such as siderophores (Bakker et al. 2014). Other studies do show differing results, such as altering a plant's SA sensitivity reducing the beneficial effects of *P. simiae* inoculation (Agorsor 2025), suggesting a direct interaction. Every strain also synthesises riboflavin, thiamin, and 2,3‐butanediol, which are established activators of plant immune defences among other functions (Shi et al. 2017; Taheri and Tarighi 2010; Lan et al. 2025; Pérez Mora et al. 2025). Riboflavin and thiamin have both been shown to enhance plant aphid defences, such as callose deposition and ROS generation, primarily through the plant's SA pathway (Tsitsekian et al. 2025; Suohui et al. 2022). Both compounds also enhance further defence responses through activation of the PAL pathway, detected to be upregulated in aphid‐resistant barley post‐bacterial inoculation (Mbaluto and Zytynska 2025). Similarly, 2,3‐butanediol activates the JA/ET and SA plant signalling pathways, depending on context, enhancing general defences such as ROS production and PR gene activation (Park et al. 2018), along with more specific defences for insect pests such as increased lignin production (Liu et al. 2023). The shared production potential for these compounds highlights that the focal strains utilise multiple redundant mechanisms, possibly to compensate for host variability or pest‐evasive techniques.

Three of the focal strains (all except the *Bacillus* spp.) possess machinery involved in *N*‐AHL quorum sensing (QS), which is linked to the induction of plant immune defences in several studies (Schikora et al. 2016; Hartmann et al. 2021). In contrast, the *Bacillus* strains instead engage in peptide‐signal‐based QS (Bareia et al. 2018; Xiong et al. 2020), which has been linked to the induction of plant JA and SA defences via lipopeptide surfactins (Farace et al. 2015; Le Mire et al. 2018). QS‐based plant responses either require QS molecules to diffuse directly into plant cells (as for short‐chain N‐AHLs associated with promoting plant growth (Sieper et al. 2014; Liu et al. 2012)) or for plants to have the ability to detect QS molecules extracellularly (as for long‐chain *N*‐AHLs linked with induction of plant immune defences (Shrestha et al. 2022; Schenk et al. 2014)). Many plants can detect microbially‐derived peptides, or their lipopeptides (Ongena et al. 2007). In addition, a plant receptor for long‐chain AHLs has been predicted, indicating these mechanisms play a key role in plant defence induction (Babenko et al. 2022). However, a previous study that knocked out AHL‐synthesis in one focal strain ( *A. radicis* ) found that this did not eliminate the aphid suppression effect, but plant transcriptional responses significantly differed (Sanchez‐Mahecha et al. 2022). This shows AHLs are not an on/off switch for these responses but may contribute to fine‐tuning of plant defences in combination with other functions.

*Acidovorax radicis* and *P. simiae* share other potential candidate mechanisms for aphid suppression, both possessing multiple biosynthetic clusters for hydrogen cyanide (HCN) and engaging in indoleacetic acid (IAA) phytohormonal pathways. Hydrogen cyanide is typically examined for its toxic effect and is synthesised by plants to help protect against herbivory (Emad Fadoul et al. 2023). It is also produced by various bacteria and thus can support the inhibition of phytopathogens and insect pests (Sehrawat et al. 2022; Kang et al. 2019). However, HCN is multifunctional in plants, serving a role as a signalling molecule for immune system activation as well as a toxin (Krasuska et al. 2023; Piekarniak et al. 2025). Rhizobacteria‐derived HCN as an inducer of plant immune defences has not yet been studied in detail, but the application of exogenous HCN to plant roots has been shown to trigger plant immune defences (Diaz‐Rueda et al. 2023). Therefore, microbial HCN is expected to have a similar effect. Both strains also display the potential to synthesise IAA or modulate plant‐synthesised IAAs through their catabolic profile. Rhizobacteria‐derived IAAs are a well‐established promoter of plant growth (Ahmed and Hasnain 2014), and have also been shown to induce plant immune defences through the ET pathway (Barnawal et al. 2017), even enhancing plant functions linked with aphid resistance, such as callose deposition (Tzipilevich et al. 2021).

Other candidate traits that are not linked to triggering immune defences are also present, with the *Bacillus* strain's broad antimicrobial profile and wide range of degradable carbon sources providing one example. Their antimicrobial arsenal is the largest among the focal strains (Stein 2005; Kiesewalter et al. 2021; Kaspar et al. 2019), supporting their established biocontrol function (Zhang et al. 2023; Jangir et al. 2018; Wang et al. 2018). Their high carbon catabolic potential also enables resilience to changing nutritional conditions, making them a potent shaper of rhizosphere composition (Lv et al. 2025; Selten and de Jonge 2025). It could be suggested that shifting the rhizosphere to a more beneficial state for the plant gives a boost to plant health, indirectly enhancing aphid suppression.

Comparing the focal strains to related pathogens reveals that many of these mechanisms are not just limited to beneficial strains, although they can be enriched depending on the genus (Tables S1 and S2). This suggests that beneficial functions may be supported by other mechanisms, such as suppression of plant‐pathogen responses through MAMP signalling (He et al. 2006) or that these strains simply lack pathogen‐related functions. *Rhizobium radiobacter* F4 aligns very closely with pathogenic strains, primarily differing only in its pTi and pAt plasmids (Glaeser et al. 2016). Future studies into this relatively small difference in genes could help establish the genomic basis for determining plant relationship status. However, the relatively small number of strains analysed limits the strength of these interpretations, and a larger set of related strains will be required to determine whether these genomic differences consistently underpin differences in plant interaction outcomes.

In conclusion, there is still much complexity in understanding how bacteria‐induced pest suppression in plants functions, but we present a rigorous genomic analysis of five aphid‐suppressive strains. Multiple candidate traits were identified, providing a robust foundation for future mechanistic investigations into the molecular basis of aphid suppression. Bacterial mechanisms that interact with plant immune defences were common across all focal strains, giving a clear indication of their importance in aphid suppression. Our study provides a valuable framework to analyse beneficial plant‐bacteria interactions, with understanding the mechanisms behind this relationship vital to fully harness bioinoculants' potential in sustainable agriculture.

## Author Contributions

**James P. J. Hall:** supervision, methodology, writing – review and editing. **Edward C. Cairns:** conceptualization, investigation, methodology, writing – original draft, visualization, writing – review and editing. **Francesco Del Carratore:** writing – review and editing, methodology, supervision. **Sharon E. Zytynska:** supervision, conceptualization, funding acquisition, methodology, writing – review and editing.

## Funding

This work was supported by UK Research and Innovation, NE/S00713X/1. Biotechnology and Biological Sciences Research Council, BB/S010556/1. Medical Research Council, MR/W02666X/1.

## Conflicts of Interest

The authors declare no conflicts of interest.

## Supporting information


**Table S1:** NCBI strain references and plant relationship status.

**Table S2:** Number of secondary metabolite biosynthetic gene clusters in each related strain. *Predicted using AntiSMASH.

**Table S3:** Number of genes in each related strain associated with plant immune system activation mechanisms. *Predicted using PLaBase.

## Data Availability

The data that support the findings of this study are openly available in GenBank NIH genetic sequence database at https://www.ncbi.nlm.nih.gov/bioproject/PRJNA1433263, reference number PRJNA1433263.
