# Supplementary material for: Revealing Functional Traits of Insect Pest Suppressive Rhizobacterial Strains Through Comparative Genomics
Source: Environ Microbiol. 2026 Sep 7;28(9):e70409. doi: 10.1111/1462-2920.70409 (PMC13550958; doi:10.1111/1462-2920.70409)
Supplement: Supplementary file 1 [file EMI-28-e70409-s001.pdf]

Strain Name	NCBI Reference	Plant Interaction	References
Acidovorax citrulli AAC00-1	CP000512.1	Pathogen	(Burdman and Walcott, 2012)
Acidovorax delafieldii 2AN	ACQT01000638.1	Neutral	NA
Acidovorax ebreus TPSY	NC_011992.1	Neutral	NA
Acidovorax facilis DSM 649	JAMXAX010000100.1	Beneficial	(Siani *et al.*, 2021)
Acidovorax sp. ST3	NZ_QKVP01000001.1	Beneficial	(Zhang et al., 2017)
Acidovorax temperans LMJ	NZ_CP117193.1	Neutral	NA
Agrobacterium radiobacter DSM 30147	NZ_CAUJMV010000001.1	Neutral	NA
Agrobacterium tumefaciens 15955	NZ_CP032917.1	Pathogen	(Holmes, Sanghera and Punja, 2023)
Agrobacterium tumefaciens A136	CP168945.1	Neutral	NA
Agrobacterium tumefaciens Ach5	NZ_CP011246.1	Pathogen	(Escobar and Dandekar, 2003)
Agrobacterium tumefaciens CCNWGS0286	NZ_AGSM01000049.1	Beneficial	(Hao *et al.*, 2012)
Agrobacterium tumefaciens LBA4213	NZ_CP007225.1	Pathogen	(Henkel *et al.*, 2014)
Bacillus altitudinis B10	JAGSNB010000001.1	Pathogen	(Lemjiber *et al.*, 2021)
Bacillus altitudinis HM-7	NZ_CP101399.1	Pathogen	(Wang *et al.*, 2022)
Bacillus pumilus 7PB	ASM1810125v1	Potential pathogen inferred from genomic traits	(Bach *et al.*, 2022)
Bacillus altitudinis strain GR-8	NZ_CP009108.1	Pathogen	(Yuan and Gao, 2015)
Bacillus amyloliquefaciens DSM 7	NC_014551.1	Neutral	NA
Bacillus atrophaeus 1942	NC_014639.1	Neutral	NA
Bacillus nakamurai NRRL B-41092	NZ_LSAZ01000001.1	Neutral	NA
Bacillus pumilus B6033	CP007436.1	Potential pathogen inferred from genomic traits	(Yuan and Gao, 2015)
Bacillus pumilus SAFR 032	CP000813.4	Potential pathogen inferred from genomic traits	(Yuan and Gao, 2015)
Bacillus subtilis BSn5	NC_014976.1	Beneficial	(Blake, Christensen and Kovács, 2021)
Bacillus subtilis PS216	NZ_AQGR01000001.1	Beneficial	(Podnar *et al.*, 2022)
Bacillus subtilis PTS-394	NZ_AWXG01000001.1	Beneficial	(Qiao *et al.*, 2017)
Bacillus subtilis subsp. subtilis NCIB 3610	CP034484.1	Beneficial	(Beauregard *et al.*, 2013)
Bacillus subtilis subsp. subtilis str. AG1839	CP008698.1	Neutral	NA
Bacillus subtilis subsp. subtilis str. BSP1	CP003695.1	Beneficial	(Hellany *et al.*, 2024)
Bacillus subtilis subsp. subtilis str. JH642	CP007800.1	Neutral	NA
Bacillus velezensis M27	AMPK01000001.1	Beneficial	(Lee *et al.*, 2012)
Bacillus velezensis NJN-6	CP007165.1	Beneficial	(Rabbee, Hwang and Baek, 2023)
Bacillus velezensis SQR9	CP006890.1	Beneficial	(Chen *et al.*, 2024)
Bacillus velezensis UCMB5033	NC_022075.1	Beneficial	(Niazi *et al.*, 2014)
Bacillus velezensis UCMB5036	NC_020410.1	Beneficial	(Manzoor *et al.*, 2013)
Bacillus velezensis YAU B9601-Y2	NC_017061.1	Beneficial	(He *et al.*, 2013)
Paracidovorax avenae ATCC 19860	CP002521.1	Pathogen	(Duan *et al.*, 2024)
Paracidovorax oryzae ATCC 19882	NZ_JMKU01000001.1	Pathogen	(Huang *et al.*, 2025)
Pseudomonas brassicacearum 3Re2-7	NZ_CP034725.1	Neutral	NA
Pseudomonas koreensis CRS05-R5	NZ_CP015852.1	Neutral	NA
Pseudomonas putida NBRC 14164	NC_021505.1	Neutral	NA
Pseudomonas savastanoi pv. phaseolicola 1448A	NC_005773.3	Neutral	NA
Pseudomonas simiae PICF7	NZ_CP005975.1	Beneficial	(Montes-Osuna *et al.*, 2022)
Pseudomonas simiae R81	NZ_JH635997.1	Beneficial	(Mathimaran *et al.*, 2012)
Pseudomonas syringae pv. tomato str. DC3000	NC_004578.1	Pathogen	(Buell *et al.*, 2003)
Pseudomonas viridiflava CFBP 1590	NZ_LT855380.1	Pathogen	(Ruinelli, Blom and Pothier, 2017)
Rhizobium pusense 76	NZ_CP053856.1	Neutral	NA
Rhizobium radiobacter SEMIA 436	NZ_JACIGT010000001.1	Beneficial	(de São José *et al.*, 2023)

Reference list

Bach, E., Rangel, C. P., Ribeiro, I. D. A. and Passaglia, L. M. P. (2022) 'Pangenome analyses of Bacillus pumilus, Bacillus safensis, and Priestia megaterium exploring the plant-associated features of bacilli strains isolated from canola', *Molecular Genetics and Genomics,* 297(4), pp. 1063–1079.

Beauregard, P. B., Chai, Y., Vlamakis, H., Losick, R. and Kolter, R. (2013) 'Bacillus subtilis biofilm induction by plant polysaccharides', *Proceedings of the national academy of sciences,* 110(17).

Blake, C., Christensen, M. N. and Kovács, Á. T. (2021) 'Molecular Aspects of Plant Growth Promotion and Protection by Bacillus subtilis', *Molecular Plant-Microbe Interactions®,* 34(1), pp. 15–25.

Buell, C. R., Joardar, V., Lindeberg, M., Selengut, J., Paulsen, I. T., Gwinn, M. L., Dodson, R. J., Deboy, R. T., Durkin, A. S., Kolonay, J. F., Madupu, R., Daugherty, S., Brinkac, L., Beanan, M. J., Haft, D. H., Nelson, W. C., Davidsen, T., Zafar, N., Zhou, L., Liu, J., Yuan, Q., Khouri, H., Fedorova, N., Tran, B., Russell, D., Berry, K., Utterback, T., Van Aken, S. E., Feldblyum, T. V., D'Ascenzo, M., Deng, W.-L., Ramos, A. R., Alfano, J. R., Cartinhour, S., Chatterjee, A. K., Delaney, T. P., Lazarowitz, S. G., Martin, G. B., Schneider, D. J., Tang, X., Bender, C. L., White, O., Fraser, C. M. and Collmer, A. (2003) 'The complete genome sequence of the Arabidopsis and tomato pathogen Pseudomonas syringae pv. tomato DC3000', *Proceedings of the national academy of sciences,* 100(18), pp. 10181–10186.

Burdman, S. and Walcott, R. O. N. (2012) 'Acidovorax citrulli: generating basic and applied knowledge to tackle a global threat to the cucurbit industry', *Molecular plant pathology,* 13(8), pp. 805–815.

Chen, Y., Li, Y., Fu, Y., Jia, L., Li, L., Xu, Z., Zhang, N., Liu, Y., Fan, X., Xuan, W., Xu, G., Zhang, R. and Gifford, M. (2024) 'The beneficial rhizobacterium Bacillus velezensis SQR9 regulates plant nitrogen uptake via an endogenous signaling pathway', *Journal of Experimental Botany,* 75(11), pp. 3388–3400.

de São José, J. F. B., Volpiano, C. G., Ambrosini, A., Lisboa, B. B., Simon, A. A., de Oliveira, J., Passaglia, L. M. P., Vargas, L. K. and Beneduzi, A. (2023) 'Indole acetic-producing bacteria promote the root development of Acacia mearnsii cuttings', *Australian Forestry,* 86(3-4), pp. 103–109.

Duan, H.-H., Zhao, J.-Y., Liu, W.-T., Xie, Q., Zheng, C.-Y. and Gao, S.-J. (2024) 'Complete genome sequence of Paracidovorax avenae causing red stripe in sugarcane', *BMC Genomic Data,* 25(1).

Escobar, M. A. and Dandekar, A. M. (2003) 'Agrobacterium tumefaciens as an agent of disease', *Trends in plant science,* 8(8), pp. 380–386.

Hao, X., Xie, P., Johnstone, L., Miller, S. J., Rensing, C. and Wei, G. (2012) 'Genome Sequence and Mutational Analysis of Plant-Growth-Promoting Bacterium Agrobacterium tumefaciens CCNWGS0286 Isolated from a Zinc-Lead Mine Tailing', *Applied and Environmental Microbiology,* 78(15), pp. 5384–5394.

He, P., Hao, K., Blom, J., Rückert, C., Vater, J., Mao, Z., Wu, Y., Hou, M., He, P., He, Y. and Borriss, R. (2013) 'Genome sequence of the plant growth promoting strain Bacillus amyloliquefaciens subsp. plantarum B9601-Y2 and expression of mersacidin and other secondary metabolites', *Journal of Biotechnology,* 164(2), pp. 281–291.

Hellany, H., Assaf, J. C., Barada, S., el-Badan, D., Hajj, R. E., Abou Najem, S., Abou Fayad, A. G. and Khalil, M. I. (2024) 'Isolation and Characterization of Bacillus Subtilis BSP1 from Soil: Antimicrobial Activity and Optimization of Fermentation Conditions', *Processes,* 12(8).

Henkel, C. V., den Dulk-Ras, A., Zhang, X. and Hooykaas, P. J. J. (2014) 'Genome Sequence of the Octopine-Type Agrobacterium tumefaciens Strain Ach5', *Genome Announcements,* 2(2).

Holmes, J. E., Sanghera, H. and Punja, Z. K. (2023) 'Crown gall development on cannabis (Cannabis sativa L., marijuana) plants caused by Agrobacterium tumefaciens species-complex', *Canadian Journal of Plant Pathology,* 45(5-6), pp. 433–445.

Huang, J., Wang, H., Zeng, Y., Guo, W., Li, F., Pan, L., Yang, S., Shan, B., Fei, N. and Zhang, X. (2025) 'First Report of Brown Stripe Disease Caused by Paracidovorax oryzae on Rice in Guangxi (China)', *Plant Disease,* 109(6).

Lee, S.-Y., Kim, B.-Y., Ahn, J.-H., Song, J., Seol, Y.-J., Kim, W.-G. and Weon, H.-Y. (2012) 'Draft Genome Sequence of the Biocontrol Bacterium Bacillus amyloliquefaciens Strain M27', *Journal of Bacteriology,* 194(24), pp. 6934–6935.

Lemjiber, N., Naamani, K., Merieau, A., Dihazi, A., Zhar, N., Jediyi, H. and Boukerb, A. M. (2021) 'Identification and Genomic Characterization of Pathogenic Bacillus altitudinis from Common Pear Trees in Morocco', *Agronomy,* 11(7).

Manzoor, S., Niazi, A., Bejai, S., Meijer, J. and Bongcam-Rudloff, E. (2013) 'Genome Sequence of a Plant-Associated Bacterium, Bacillus amyloliquefaciens Strain UCMB5036', *Genome Announcements,* 1(2).

Mathimaran, N., Srivastava, R., Wiemken, A., Sharma, A. K. and Boller, T. (2012) 'Genome Sequences of Two Plant Growth-Promoting Fluorescent Pseudomonas Strains, R62 and R81', *Journal of Bacteriology,* 194(12), pp. 3272–3273.

Montes-Osuna, N., Cernava, T., Gómez-Lama Cabanás, C., Berg, G. and Mercado-Blanco, J. (2022) 'Identification of Volatile Organic Compounds Emitted by Two Beneficial Endophytic Pseudomonas Strains from Olive Roots', *Plants,* 11(3).

Niazi, A., Manzoor, S., Bejai, S., Meijer, J. and Bongcam-Rudloff, E. (2014) 'Complete genome sequence of a plant associated bacterium Bacillus amyloliquefaciens subsp. plantarum UCMB5033', *Standards in Genomic Sciences,* 9(3), pp. 718–725.

Podnar, E., Erega, A., Danevčič, T., Kovačec, E., Lories, B., Steenackers, H., Mandic-Mulec, I., Kolodkin-Gal, I., Chai, Y. and Barbieri, G. (2022) 'Nutrient Availability and Biofilm Polysaccharide Shape the Bacillaene-Dependent Antagonism of Bacillus subtilis against Salmonella Typhimurium', *Microbiology Spectrum,* 10(6).

Qiao, J., Yu, X., Liang, X., Liu, Y., Borriss, R. and Liu, Y. (2017) 'Addition of plant-growth-promoting Bacillus subtilis PTS-394 on tomato rhizosphere has no durable impact on composition of root microbiome', *BMC Microbiology,* 17(1).

Rabbee, M. F., Hwang, B.-S. and Baek, K.-H. (2023) 'Bacillus velezensis: A Beneficial Biocontrol Agent or Facultative Phytopathogen for Sustainable Agriculture', *Agronomy,* 13(3).

Ruinelli, M., Blom, J. and Pothier, J. F. (2017) 'Complete Genome Sequence of Pseudomonas viridiflava CFBP 1590, Isolated from Diseased Cherry in France', *Genome Announcements,* 5(30).

Siani, R., Stabl, G., Gutjahr, C., Schloter, M. and Radl, V. (2021) 'Acidovorax pan-genome reveals specific functional traits for plant beneficial and pathogenic plant-associations', *Microbial Genomics,* 7(12).

Wang, Q., Zhang, L., Zhang, Y., Chen, H., Song, J., Lyu, M., Chen, R. and Zhang, L. (2022) 'Comparative genomic analyses reveal genetic characteristics and pathogenic factors of Bacillus pumilus HM-7', *Frontiers in Microbiology,* 13.

Yuan, Y. and Gao, M. (2015) 'Genomic analysis of a ginger pathogen Bacillus pumilus providing the understanding to the pathogenesis and the novel control strategy', *Scientific Reports,* 5(1).

Zhang, J., Zhao, S., Xu, Y., Zhou, W., Huang, K., Tang, Z. and Zhao, F.-J. (2017) 'Nitrate Stimulates Anaerobic Microbial Arsenite Oxidation in Paddy Soils', *Environmental Science & Technology,* 51(8), pp. 4377–4386.
