# Supplementary material for: Revealing Functional Traits of Insect Pest Suppressive Rhizobacterial Strains Through Comparative Genomics
Source: Environ Microbiol. 2026 Sep 7;28(9):e70409. doi: 10.1111/1462-2920.70409 (PMC13550958; doi:10.1111/1462-2920.70409)
Supplement: Supplementary file 2 [file EMI-28-e70409-s002.pdf]

	ISR-ACETOIN |2|3-BUTANEDIOL BIOSYNTHESIS	ISR-AHL|NAHL BIOSYNTHESIS	ISR-ISOJASMONIC ACID BIOSYNTHESIS	ISR-VITAMIN B1|THIAMIN BIOSYNTHESIS	ISR-VITAMIN B1|THIAMIN TRANSPORT	ISR-VITAMIN B2|RIBOFLAVIN BIOSYNTHESIS	PTI-AVRXA21|XA21	PTI-BACTERIAL EF-TU	PTI-FLAGELLIN TRIGGERED IMMUNITY	PTI-NOD FACTORS	PTI-ORNITHINE LIPIDS BIOSYNTHESIS	PTI-PR1 LIKE PROTEINS	PTI-SURFACTIN PRODUCTION	PTI-TEICHURONIC ACID|LPS ELICITOR	PTI-XOPD LIKE PROTEINS	SAR-SALICYLIC ACID BIOSYNTHESIS	SAR-PIPECOLATE BIOSYNTHESIS	PTI-EPS YDALMN	PTI-SALIC ACID BIOSYNTHESIS	ISR-ENTEROBACTIN|ENTEROCHELIN	SAR-SALICYLIC ACID TRANSPORT
Acidovorax citrulli AAC00-1	18	6	2	16	1	13	1	3	8	6	2	1	2	4	2	3	0	0	0	0	0
Acidovorax delafieldii 2AN	17	0	2	16	1	15	1	3	8	4	2	1	0	7	0	2	2	0	0	0	0
Acidovorax ebreus TPSY	15	1	2	17	0	17	1	3	8	5	2	1	0	5	0	2	2	1	0	0	0
Acidovorax facilis DSM 649	17	6	2	16	1	11	1	3	8	7	2	1	0	7	0	3	0	2	0	0	0
Acidovorax radicis N35	18	5	2	16	1	13	1	3	8	6	2	1	0	6	0	2	0	2	0	0	0
Acidovorax sp. ST3	17	1	2	16	1	13	2	3	8	6	2	1	0	3	0	6	0	2	0	0	0
Acidovorax temperans LMJ	18	0	2	17	1	13	1	3	8	6	1	1	0	3	0	2	0	0	0	0	0
Agrobacterium radiobacter DSM 30147	18	1	2	18	4	16	0	3	6	3	3	0	0	6	0	2	2	2	2	0	0
Agrobacterium tumefaciens 15955	19	1	2	19	4	15	0	3	6	2	3	0	0	6	0	9	2	2	0	8	0
Agrobacterium tumefaciens A136	19	0	2	18	4	15	0	3	6	2	3	0	0	6	0	3	2	2	0	0	0
Agrobacterium tumefaciens Ach5	18	1	2	19	4	13	0	3	6	2	3	0	0	6	0	8	2	2	0	8	0
Agrobacterium tumefaciens CCNWGS0286	16	1	2	18	4	16	0	3	6	2	3	0	0	3	0	2	2	2	0	0	0
Agrobacterium tumefaciens LBA4213	18	1	2	19	4	13	0	3	6	2	3	0	0	6	0	8	2	2	0	8	0
Bacillus altitudinis B10	23	0	2	23	1	16	0	3	8	0	0	0	9	13	0	6	0	4	1	8	0
Bacillus altitudinis HM-7	23	0	2	23	1	15	0	3	8	0	0	0	9	13	0	0	0	4	1	0	0
Bacillus altitudinis strain GR-8	23	0	2	23	1	17	0	3	8	0	0	0	9	13	0	0	0	4	1	0	0
Bacillus amyloliquefaciens DSM 7	24	0	2	24	2	22	0	3	8	0	0	1	9	13	0	6	0	4	1	8	0
Bacillus atrophaeus 1942	24	0	2	24	2	23	0	3	8	0	0	1	9	13	0	6	0	4	0	8	0
Bacillus nakamurai NRRL B-41092	26	0	2	24	2	21	0	3	8	0	0	1	7	13	0	6	0	4	1	8	0
Bacillus pumilus 7PB	23	0	2	23	1	18	0	3	8	1	0	0	9	13	0	6	0	4	1	8	0
Bacillus pumilus B6033	23	0	2	23	1	17	0	3	8	0	0	0	9	13	0	0	0	4	1	0	0
Bacillus pumilus SAFR-032	23	0	2	23	1	18	0	3	8	1	0	0	9	13	0	0	0	4	1	0	0
Bacillus subtilis B171	24	0	2	24	2	21	0	3	8	0	0	1	9	13	0	6	0	4	1	8	0
Bacillus subtilis BSn5	24	0	2	24	2	21	0	3	8	0	0	1	9	13	0	6	0	4	1	8	0
Bacillus subtilis PS216	24	0	2	24	2	21	0	3	8	0	0	1	9	13	0	6	0	4	1	8	0
Bacillus subtilis PTS-394	24	0	2	24	2	21	0	3	8	0	0	1	10	13	0	6	0	4	1	8	0
Bacillus subtilis subsp. subtilis NCIB 3610	24	0	2	24	2	21	0	3	8	0	0	1	9	13	0	6	0	4	1	8	0
Bacillus subtilis subsp. subtilis str. AG1839	24	0	2	24	2	21	0	3	8	0	0	1	9	13	0	6	0	0	1	8	0
Bacillus subtilis subsp. subtilis str. BSP1	24	0	2	24	2	21	0	3	8	0	0	1	9	13	0	6	0	4	1	8	0
Bacillus subtilis subsp. subtilis str. JH642	24	0	2	24	2	21	0	3	8	0	0	1	9	13	0	6	0	0	1	8	0
Bacillus velezensis FZB42	24	0	2	24	2	22	0	3	8	0	0	1	9	13	0	7	0	4	1	8	0
Bacillus velezensis M27	24	0	2	24	2	22	0	3	8	0	0	1	9	13	0	6	0	4	1	8	0
Bacillus velezensis NJN-6	24	0	2	24	2	22	0	3	8	0	0	1	9	13	0	7	0	4	1	8	0
Bacillus velezensis SQR9	24	0	2	24	2	22	0	3	8	0	0	1	9	13	0	6	0	4	1	8	0
Bacillus velezensis UCMB5033	24	0	2	24	2	22	0	3	8	0	0	1	9	13	0	6	0	4	1	8	0
Bacillus velezensis UCMB5036	24	0	2	24	2	22	0	3	8	0	0	1	9	13	0	6	0	4	1	8	0
Bacillus velezensis YAU B9601-Y2	24	0	2	24	2	22	0	3	8	0	0	1	7	13	0	6	0	4	1	8	0
Paracidovorax avenae ATCC 19860	19	1	2	16	1	15	0	3	8	4	2	1	2	3	2	2	0	0	0	0	0
Paracidovorax oryzae ATCC 19882	19	1	2	16	1	17	1	3	8	7	2	1	2	4	0	2	0	0	0	0	0
PseudomonassimiaeWCS417r	19	1	2	16	1	16	1	3	8	6	3	1	0	5	0	11	2	0	0	0	1
Pseudomonas brassicacearum 3Re2-7	18	1	2	16	1	16	2	3	8	5	2	1	0	7	0	2	2	0	0	0	1
Pseudomonas koreensis CRS05-R5	16	1	2	17	1	15	1	3	8	4	2	1	2	6	0	3	2	0	1	0	0
Pseudomonas putida NBRC 14164	20	1	2	18	1	21	1	3	8	5	2	1	0	5	0	2	2	2	1	0	0
Pseudomonas savastanoi pv. phaseolicola 1448A	15	3	2	16	1	17	5	3	8	2	2	1	2	5	0	4	2	0	0	0	0
Pseudomonas simiae PICF7	20	1	2	16	1	16	1	3	8	6	3	1	0	5	0	11	2	0	0	0	1
Pseudomonas simiae R81	19	1	2	16	1	16	1	3	8	6	3	1	0	5	0	11	2	0	0	0	1
Pseudomonas simiae WCS417r	19	1	2	16	1	16	1	3	8	6	3	1	0	5	0	11	2	0	0	0	1
Pseudomonas syringae pv. tomato str. DC3000	17	3	2	16	1	17	1	3	8	2	2	1	2	5	0	4	2	2	0	0	0
Pseudomonas viridiflava CFBP 1590	17	1	2	15	1	15	1	3	8	5	2	1	2	5	0	0	2	0	0	0	0
Rhizobium pusense 76	16	0	2	18	4	16	0	3	6	2	3	0	0	3	0	8	2	2	0	8	0
Rhizobium radiobacter F4	19	1	2	18	4	15	0	3	6	2	3	0	0	6	0	3	2	2	0	0	0
Rhizobium radiobacter SEMIA 436	18	1	2	20	4	16	0	3	6	3	3	0	0	6	0	2	2	2	2	0	0
