# Supplementary material for: Revealing Functional Traits of Insect Pest Suppressive Rhizobacterial Strains Through Comparative Genomics
Source: Environ Microbiol. 2026 Sep 7;28(9):e70409. doi: 10.1111/1462-2920.70409 (PMC13550958; doi:10.1111/1462-2920.70409)
Supplement: Supplementary file 3 [file EMI-28-e70409-s003.pdf]

	Acyl amino acids	Arylpolyene	Azole-containing-RiPP	Betalactone	Butyrolactone	CDPS	Epipeptide	Furan	Glycocin	HR-T2PKS	Hserlactone	Hydrogen-cyanide	Lanthipeptide-class-i	Lanthipeptide-class-ii	Lanthipeptide-class-iii	Lassopeptide	NAGGN	NAPAA	NI-siderophore	NRP-metallophore	NRPS	NRPS-like	Other	Phosphonate	Phosphonate-like	PKS-like	Ranthipeptide	Redox-cofactor	RiPP-like	RRE-containing	Sactipeptide	T1PKS	T3PKS	Phytoene/Squalene-Synthase	Terpene	Terpene-precursor	Thioamitides	TransAT-PKS	TransAT-PKS-like
**Acidovorax citrulli AAC00-1**	0	0	0	1	0	0	0	0	0	0	1	0	0	0	0	0	0	0	0	1	2	1	0	0	1	0	0	0	1	0	0	1	0	1	0	1	0	0	0
**Acidovorax delafieldii 2AN**	0	0	0	0	0	0	0	0	0	0	0	2	0	0	0	0	0	0	0	0	0	0	0	0	0	0	0	0	0	0	0	0	0	1	0	0	0	0	0
**Acidovorax ebreus TPSY**	0	0	0	2	0	0	0	0	0	0	0	0	0	0	0	0	0	0	0	0	0	0	0	0	0	0	0	0	0	0	0	0	0	1	0	1	0	0	0
**Acidovorax facilis DSM 649**	1	0	0	0	0	0	0	0	0	0	2	1	0	0	0	0	0	0	0	2	2	2	0	0	0	0	0	0	2	0	0	1	0	1	0	1	0	0	0
**Acidovorax radicis N35**	1	0	0	2	0	0	0	0	0	0	1	2	0	0	0	0	0	0	1	0	0	0	0	0	0	0	0	0	1	0	0	0	0	1	0	1	0	0	0
**Acidovorax sp. ST3**	0	0	0	0	0	0	0	0	0	0	0	3	0	0	0	0	0	0	0	0	0	0	0	0	0	0	0	0	2	0	0	0	0	1	0	1	0	0	0
**Acidovorax temperans LMJ**	0	0	0	2	0	0	0	0	0	0	0	0	0	0	0	0	0	0	0	0	0	0	0	0	0	0	0	0	0	0	0	0	0	1	0	1	0	0	0
**Agrobacterium radiobacter DSM 30147**	0	0	0	0	0	0	0	0	0	0	2	1	0	0	0	0	0	0	0	1	1	0	0	0	0	0	0	0	0	0	0	0	0	1	0	1	1	0	0
**Agrobacterium tumefaciens 15955**	0	0	0	0	0	0	0	0	0	0	3	1	0	0	0	0	0	0	0	2	2	0	0	0	0	0	0	0	0	0	0	1	0	1	0	1	1	0	0
**Agrobacterium tumefaciens A136**	0	0	0	0	0	0	0	0	0	0	0	0	0	0	0	0	0	1	0	1	1	0	0	0	0	0	0	0	0	0	0	1	0	1	0	1	1	0	0
**Agrobacterium tumefaciens Ach5**	0	0	0	0	0	0	0	0	0	0	1	1	0	0	0	0	0	0	0	1	1	0	0	0	0	0	0	0	0	0	0	0	0	1	0	1	1	0	0
**Agrobacterium tumefaciens CCNWGS0286**	0	0	0	0	0	0	0	0	0	0	1	1	0	0	0	0	0	0	0	1	1	0	0	0	0	0	0	0	0	0	0	0	0	1	0	1	1	0	0
**Agrobacterium tumefaciens LBA4213**	0	0	0	0	0	0	0	0	0	0	1	1	0	0	0	0	0	0	0	1	1	0	0	0	0	0	0	0	0	0	0	0	0	1	0	1	1	0	0
**Bacillus altitudinis B10**	0	0	0	2	0	0	0	0	0	0	0	0	0	0	0	0	0	0	1	1	4	0	1	0	0	0	0	0	2	1	0	0	1	1	1	1	0	0	0
**Bacillus altitudinis HM-7**	0	0	0	2	0	0	0	0	0	0	0	0	0	0	0	0	0	0	1	0	1	0	1	0	0	0	0	0	1	1	0	0	1	1	1	1	0	0	0
**Bacillus altitudinis strain GR-8**	0	0	0	2	0	0	0	0	0	0	0	0	0	0	0	0	0	0	1	0	1	0	1	0	0	0	0	0	1	1	0	0	1	1	1	2	0	0	0
**Bacillus amyloliquefaciens DSM 7**	0	0	0	1	0	0	0	0	0	0	0	0	0	0	1	0	0	0	0	1	5	0	1	0	0	1	0	0	1	0	0	0	2	1	1	2	0	2	0
**Bacillus atrophaeus 1942**	0	0	1	1	0	0	0	0	0	1	0	0	0	0	0	0	0	0	0	1	5	0	0	0	0	1	0	0	1	0	1	0	2	1	1	2	0	2	0
**Bacillus nakamurai NRRL B-41092**	0	0	0	1	0	0	0	0	0	1	0	0	0	0	1	0	0	0	0	1	4	1	1	0	0	1	0	0	1	0	0	0	1	1	1	1	0	2	0
**Bacillus pumilus 7PB**	0	0	0	2	0	0	0	0	0	0	0	0	0	0	0	0	0	0	1	1	3	0	1	0	0	0	0	0	1	1	1	1	1	1	1	2	0	0	0
**Bacillus pumilus B6033**	0	0	0	2	0	0	0	0	0	0	0	0	0	0	0	0	0	0	1	0	1	1	1	0	0	0	0	0	2	1	0	0	1	1	1	2	0	0	0
**Bacillus pumilus SAFR 032**	0	0	0	2	0	0	0	0	0	0	0	0	0	0	0	0	0	0	1	0	2	0	1	0	0	0	0	0	1	1	0	1	1	1	1	2	0	0	0
**Bacillus subtilis B171**	0	0	0	1	0	1	1	0	0	0	0	0	0	0	0	0	0	0	0	1	4	0	1	0	0	1	1	0	0	0	2	0	2	1	1	2	0	1	0
**Bacillus subtilis BSn5**	0	0	0	1	0	0	0	0	0	0	0	0	1	0	0	0	0	0	0	1	4	1	1	0	0	1	0	0	0	0	1	0	2	1	1	2	0	1	0
**Bacillus subtilis PS216**	0	0	0	1	0	1	1	0	0	0	0	0	0	0	0	0	0	0	0	1	7	0	1	0	0	1	1	0	0	1	3	0	2	1	1	2	0	1	0
**Bacillus subtilis PTS-394**	0	0	0	1	0	1	0	0	0	0	0	0	0	0	1	0	0	0	0	1	6	0	1	0	0	1	0	0	0	1	1	0	2	1	1	2	0	1	0
**Bacillus subtilis subsp. subtilis NCIB 3610**	0	0	0	1	0	1	1	0	1	0	0	0	0	0	0	0	0	0	0	1	4	0	1	0	0	1	1	0	0	1	2	0	2	1	1	2	0	1	0
**Bacillus subtilis subsp. subtilis str. AG1839**	0	0	0	1	0	1	1	0	1	0	0	0	0	0	0	0	0	0	0	1	4	0	1	0	0	1	1	0	0	1	2	0	2	1	1	2	0	1	0
**Bacillus subtilis subsp. subtilis str. BSP1**	0	0	0	1	0	1	0	0	0	0	0	0	0	0	0	0	0	0	0	1	4	0	1	0	0	1	1	0	0	0	2	0	2	1	1	2	0	1	0
**Bacillus subtilis subsp. subtilis str. JH642**	0	0	0	1	0	1	1	0	1	0	0	0	0	0	0	0	0	0	0	1	4	0	1	0	0	1	1	0	0	1	2	0	2	1	1	2	0	1	0
**Bacillus velezensis FZB42**	0	0	1	1	0	0	0	0	0	0	0	0	0	0	0	0	0	0	0	1	5	0	1	0	0	1	0	0	1	1	0	0	2	1	1	1	0	4	0
**Bacillus velezensis M27**	0	0	0	1	0	0	0	0	0	0	0	0	0	1	0	0	0	0	0	1	7	0	1	0	0	1	0	0	1	0	0	0	2	1	1	2	0	4	0
**Bacillus velezensis NJN-6**	0	0	0	1	0	0	0	0	0	0	0	0	0	1	0	0	0	0	0	1	5	0	1	0	0	1	0	0	1	0	0	0	2	1	1	2	0	5	0
**Bacillus velezensis SQR9**	0	0	0	1	0	0	0	0	0	0	0	0	0	0	0	0	0	0	0	1	4	0	1	0	0	1	0	0	1	0	0	1	3	1	1	2	0	5	0
**Bacillus velezensis UCMB5033**	0	0	0	1	0	0	0	0	0	0	0	0	0	0	0	0	0	0	0	1	5	1	1	0	0	1	0	0	1	0	0	0	2	1	1	2	0	4	1
**Bacillus velezensis UCMB5036**	0	0	0	1	0	0	0	0	0	0	0	0	0	0	0	0	0	0	0	1	5	0	1	0	0	1	0	0	1	0	0	0	2	1	1	2	0	4	0
**Bacillus velezensis YAU B9601-Y2**	0	0	0	1	0	0	0	0	0	0	0	0	0	1	0	0	0	0	0	1	5	0	1	1	0	1	0	0	1	0	0	0	2	1	1	2	0	4	0
**Paracidovorax avenae ATCC 19860**	0	1	0	1	0	0	0	0	0	0	0	0	0	0	0	1	0	0	0	1	2	1	0	1	0	0	0	0	1	0	0	1	0	1	0	1	0	0	0
**Paracidovorax oryzae ATCC 19882**	0	0	0	1	0	0	0	0	0	0	0	0	0	0	0	1	0	0	0	1	2	1	0	0	0	0	0	0	1	0	0	1	0	1	0	1	0	0	0
**Pseudomonas brassicacearum 3Re2-7**	0	1	0	1	1	0	0	0	0	0	0	4	0	0	0	0	1	0	0	2	3	1	0	0	0	0	0	1	1	0	0	0	1	0	0	1	0	0	0
**Pseudomonas koreensis CRS05-R5**	0	1	0	1	0	0	0	0	0	0	0	1	0	0	0	0	1	0	0	1	3	1	0	0	0	0	0	1	3	0	0	0	0	0	0	1	0	0	0
**Pseudomonas putida NBRC 14164**	0	0	0	0	0	0	0	0	0	0	0	0	0	0	0	0	1	0	0	2	2	0	0	0	0	0	1	1	3	0	0	0	0	0	0	1	0	0	0
**Pseudomonas savastanoi pv. phaseolicola 1448A**	0	1	0	0	0	0	0	1	0	0	1	2	0	0	0	0	1	1	1	2	4	2	0	0	0	0	0	1	0	0	0	1	0	0	0	1	0	0	0
**Pseudomonas simiae PICF7**	0	1	0	1	0	0	0	0	0	0	0	1	0	0	0	0	1	0	0	2	3	1	0	0	0	0	0	1	5	0	0	0	0	0	1	1	0	0	0
**Pseudomonas simiae R81**	1	0	1	0	0	0	0	0	0	0	1	0	0	0	0	1	0	0	2	3	1	0	0	0	0	0	1	5	0	0	0	0	0	1	1	0	0	0	0
**Pseudomonas simiae WCS417r**	0	1	0	1	0	0	0	0	0	0	0	1	0	0	0	0	1	0	0	2	3	1	0	0	0	0	0	1	5	0	0	0	0	0	1	1	0	0	0
**Pseudomonas syringae pv. tomato str. DC3000**	1	0	0	0	0	0	0	0	0	1	2	0	0	0	0	1	0	0	2	5	2	0	0	0	0	0	1	0	0	0	2	0	0	0	1	0	0	0	0
**Pseudomonas viridiflava CFBP 1590**	1	0	0	0	0	0	0	0	0	0	2	0	0	0	0	1	1	1	1	3	1	0	0	0	0	0	1	0	0	0	0	0	0	1	1	0	0	0	0
**Rhizobium pusense 76**	0	0	0	0	0	0	0	0	0	0	0	0	0	0	0	0	0	0	1	1	0	0	0	0	0	0	0	0	0	0	0	0	1	0	1	1	0	0	0
**Rhizobium radiobacter F4**	0	0	0	0	0	0	0	0	0	1	0	0	0	0	0	0	1	0	1	1	0	0	0	0	0	0	0	0	0	0	1	0	1	0	1	1	0	0	0
**Rhizobium radiobacter SEMIA 436**	0	0	0	0	0	0	0	0	0	2	1	0	0	0	0	0	0	0	1	1	0	0	0	0	0	0	0	0	0	0	0	0	1	0	1	1	0	0	0
